# Supplementary material for: Flavanonol Glycosides from the Stems of Myrsine seguinii and Their Neuroprotective Activities
Source: Pharmaceuticals (Basel). 2021 Sep 9;14(9):911. doi: 10.3390/ph14090911 (PMC8472518; doi:10.3390/ph14090911)
Supplement: Supplementary file 1 [file pharmaceuticals-14-00911-s001.zip › pharmaceuticals-1348732-supplementary.pdf]

Supplementary Materials

Flavanonol Glycosides from the Stems of *Myrsine seguinii* and their Neuroprotective Activities

Hee Ju Lee [1,2], Eun Jin Park [1], Ba Wool Lee [1], Hyo Moon Cho [1], Thi-Linh-Giang Pham [3], Quynh-Hoa Hoang [3], Cheol-Ho Pan [2] and Won Keun Oh [1,*]

[1] Korea Bioactive Natural Material Bank, Research Institute of Pharmaceutical Sciences, College of Pharmacy, Seoul National University, Seoul 08826, Republic of Korea

[2] Natural Product Informatics Research Center, Korea Institute of Science and Technology, Gangneung, 25451, Republic of Korea

[3] Department of Botany, Hanoi University of Pharmacy, Hanoi, Vietnam

[*] Correspondence: wkoh1@snu.ac.kr (W.K.O.)

Table of Contents

Figure S1. The neighbor-joining tree based on four DNA sequences.

Figure S2. Molecular network of extract and fractions from *M. seguinii.*

Figure S3. HR-ESI-MS of compound **1**.

Figure S4. IR sptectrum of compound **1**.

Figure S5. ^{1}H NMR spectrum (CD_3OD, 400 MHz) of compound **1**.

Figure S6. ^{13}C NMR spectrum (CD_3OD, 100 MHz) of compound **1**.

Figure S7. HSQC spectrum (CD_3OD, 400 MHz) of compound **1.**

Figure S8. HMBC spectrum (CD_3OD, 400 MHz) of compound **1**.

Figure S9. ^{1}H-^{1}H COSY spectrum (CD_3OD, 400 MHz) of compound **1**.

Figure S10. HR-ESI-MS of compound **2**.

Figure S11. IR sptectrum of compound **2**.

Figure S12. ^{1}H NMR spectrum (CD_3OD, 400 MHz) of compound **2**.

Figure S13. ^{13}C NMR spectrum (CD_3OD, 100 MHz) of compound **2**.

Figure S14. HSQC spectrum (CD_3OD, 400 MHz) of compound **2**.

Figure S15. HMBC spectrum (CD_3OD, 400 MHz) of compound **2**.

Figure S16. ^{1}H-^{1}H COSY spectrum (CD_3OD, 400 MHz) of compound **2**.

Figure S17. HR-ESI-MS of compound **3**.

Figure S18. IR sptectrum of compound **3**.

Figure S19. ^{1}H NMR spectrum (CD_3OD, 400 MHz) of compound **3**.

Figure S20. ^{13}C NMR spectrum (CD_3OD, 100 MHz) of compound **3**.

Figure S21. HSQC spectrum (CD_3OD, 400 MHz) of compound **3**.

Figure S22. HMBC spectrum (CD_3OD, 400 MHz) of compound **3**.

Figure S23. ^{1}H-^{1}H COSY spectrum (CD_3OD, 400 MHz) of compound **3.**

Figure S24. HR-ESI-MS of compound **4**.

Figure S25. IR sptectrum of compound **4**.

Figure S26. ^{1}H NMR spectrum (CD_3OD, 500 MHz) of compound **4**.

Figure S27. ^{13}C NMR spectrum (CD_3OD, 125 MHz) of compound **4**.

Figure S28. HSQC spectrum (CD_3OD, 500 MHz) of compound **4**.

Figure S29. HMBC spectrum (CD_3OD, 500 MHz) of compound **4**.

Figure S30. ^{1}H-^{1}H COSY spectrum (CD_3OD, 500 MHz) of compound **4**.

Figure S31. HR-ESI-MS of compound **5**.

Figure S32. IR sptectrum of compound **5**.

Figure S33. ^{1}H NMR spectrum (CD_3OD, 400 MHz) of compound **5**.

Figure S34. ^{13}C NMR spectrum (CD_3OD, 100 MHz) of compound **5**.

Figure S35. HSQC spectrum (CD_3OD, 400 MHz) of compound **5**.

Figure S36. HMBC spectrum (CD_3OD, 400 MHz) of compound **5**.

Figure S37. ^{1}H-^{1}H COSY spectrum (CD_3OD, 400 MHz) of compound **5**.

Figure S38. HR-ESI-MS of compound **6**.

Figure S39. IR sptectrum of compound **6**.

Figure S40. ^{1}H NMR spectrum (CD_3OD, 500 MHz) of compound **6**.

Figure S41. ^{13}C NMR spectrum (CD_3OD, 125 MHz) of compound **6**.

Figure S42. HSQC spectrum (CD_3OD, 500 MHz) of compound **6**.

Figure S43. HMBC spectrum (CD_3OD, 500 MHz) of compound **6**.

Figure S44. ^{1}H-^{1}H COSY spectrum (CD_3OD, 500 MHz) of compound **6**.

Figure S45. HR-ESI-MS of compound **7**.

Figure S46. IR sptectrum of compound **7**.

Figure S47. ^{1}H NMR spectrum (CD_3OD, 800 MHz) of compound **7**.

Figure S48. ^{13}C NMR spectrum (CD_3OD, 200 MHz) of compound **7**.

Figure S49. HSQC spectrum (CD_3OD, 800 MHz) of compound **7**.

Figure S50. HMBC spectrum (CD_3OD, 800 MHz) of compound **7**.

Figure S51. ^1H-^1H COSY spectrum (CD_3OD, 800 MHz) of compound **7.**

Figure S52. Experimental circular dichroism (ECD) spectra of compounds **1**–**7**.

Figure S53. Cytotoxicity of compounds **1**–**12** in HT22 cells.

Figure S54. Cell morphology of HT22 cells treated with compounds **1**–**12** after pEGFP-C1/A$\beta_{1\text{-}42}$ plasmid transfection.

Table S1. BLAST results of *M. seguinii*.

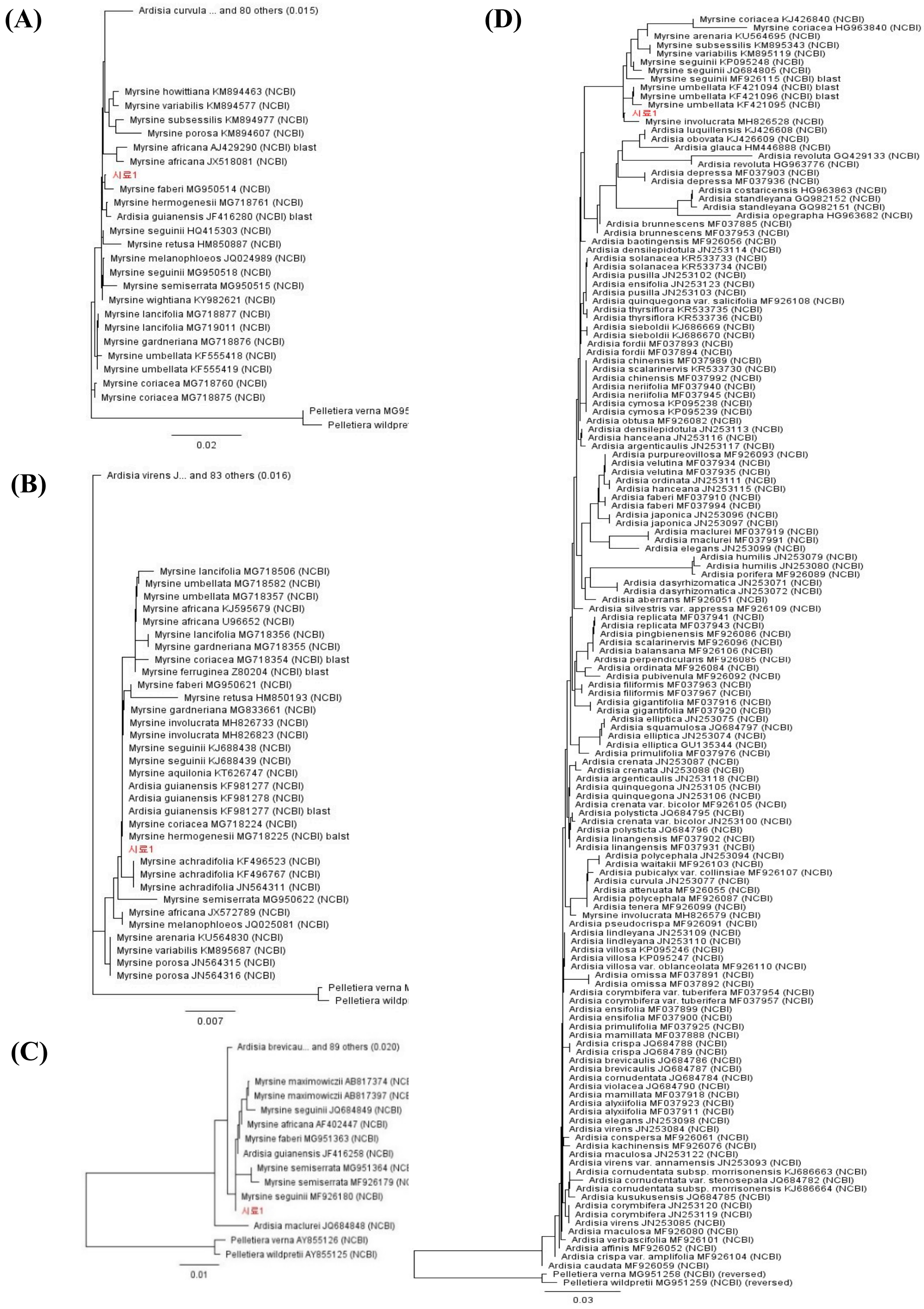

Figure S1. The neighbor-joining tree based on four DNA sequences. (A) *mat*K sequences; (B) *rbc*L sequences; (C) *trn*L-*trn*F sequences; (D) *trn*H-*psb*A sequences. The red character was directly sequenced in this study, and the other sample sequences were downloaded from GenBank via Blast analysis.

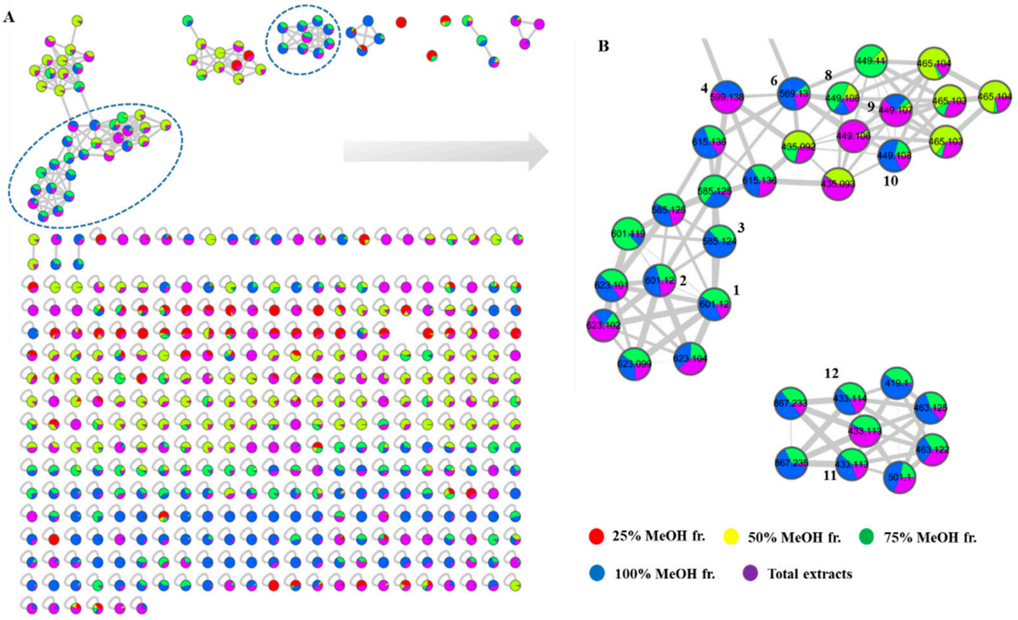

Figure S2. Molecular network of extract and fractions from *M. seguinii*. (A) Full clusters of *M. seguinii* extract and fractions; (B) Targeted cluster of flavanone glycosides derivatives in molecular networking.

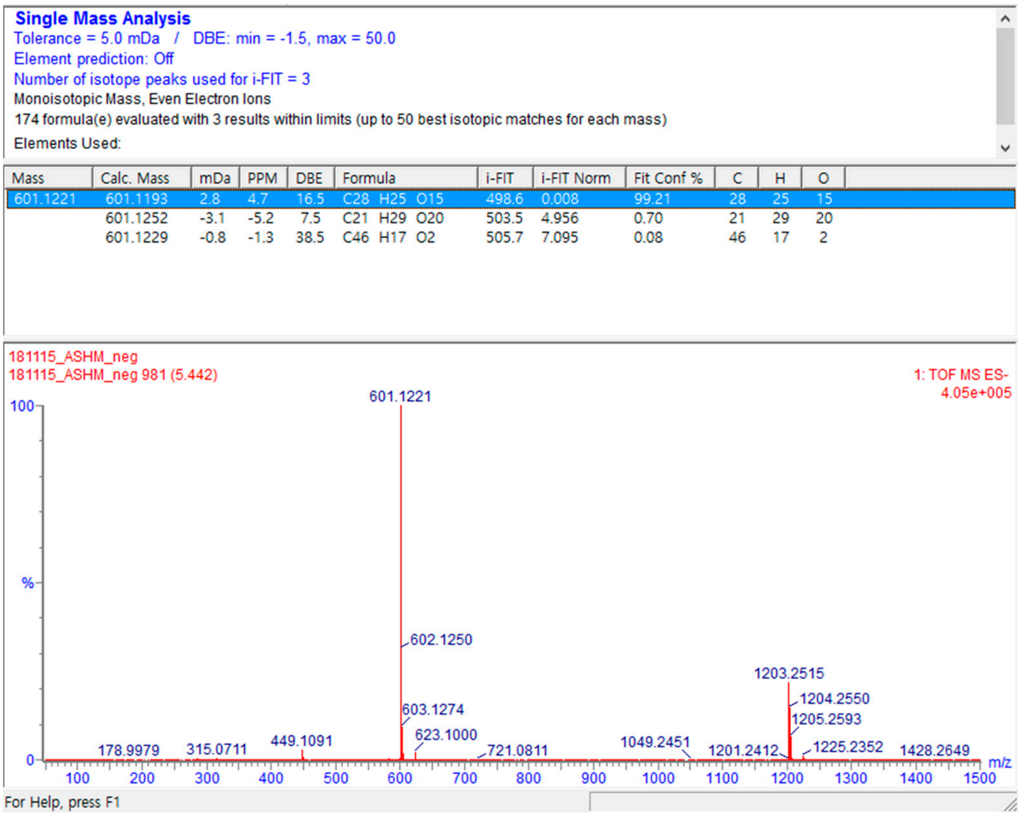

Figure S3. HR-ESI-MS of compound **1**.

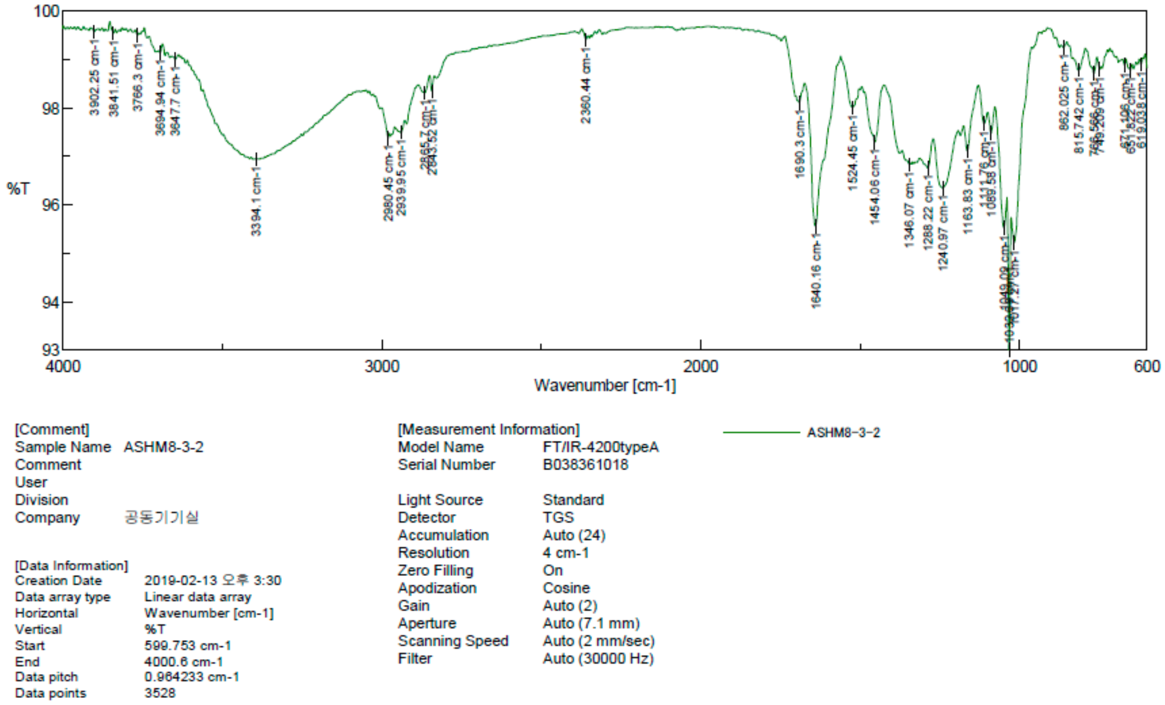

Figure S4. IR spectrum of compound **1**.

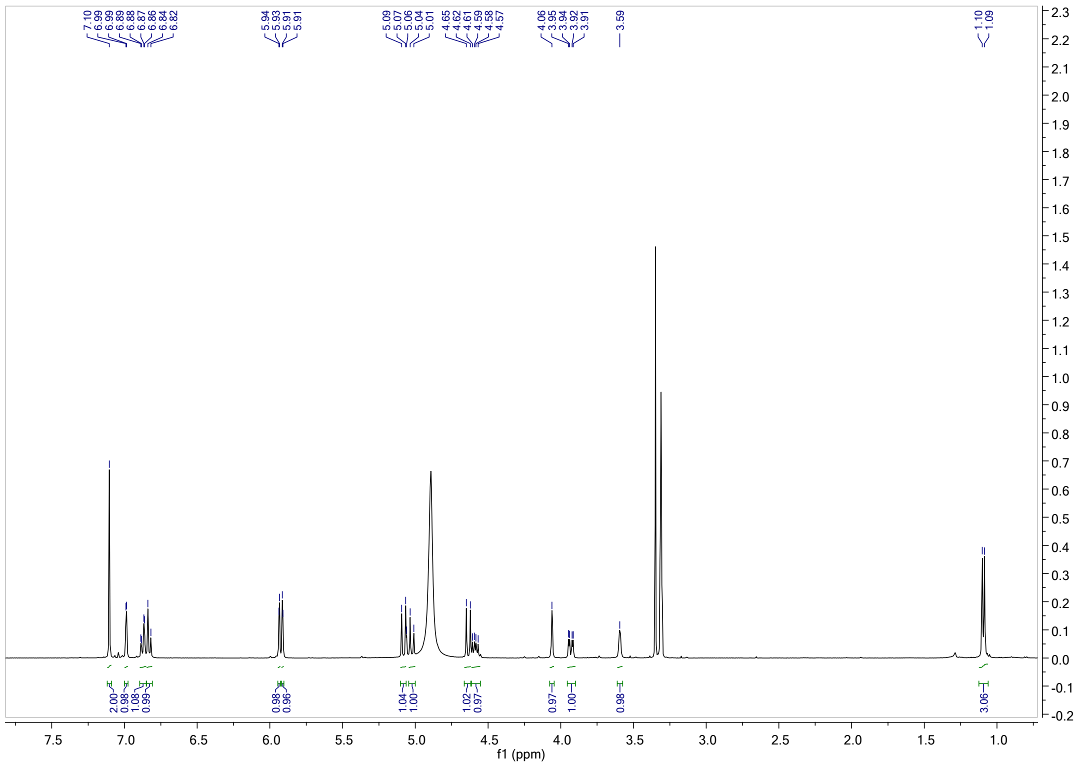

Figure S5. ^{1}H NMR spectrum (CD_3OD, 400 MHz) of compound **1**.

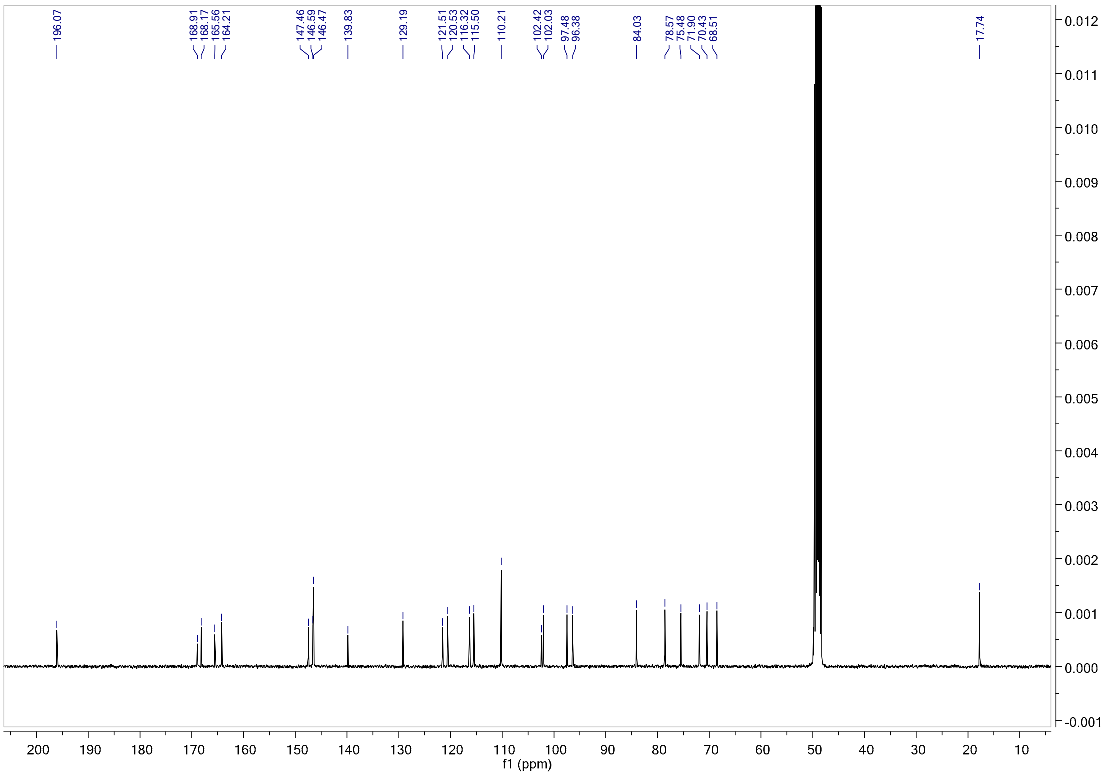

Figure S6. ^{13}C NMR spectrum (CD_3OD, 100 MHz) of compound **1**.

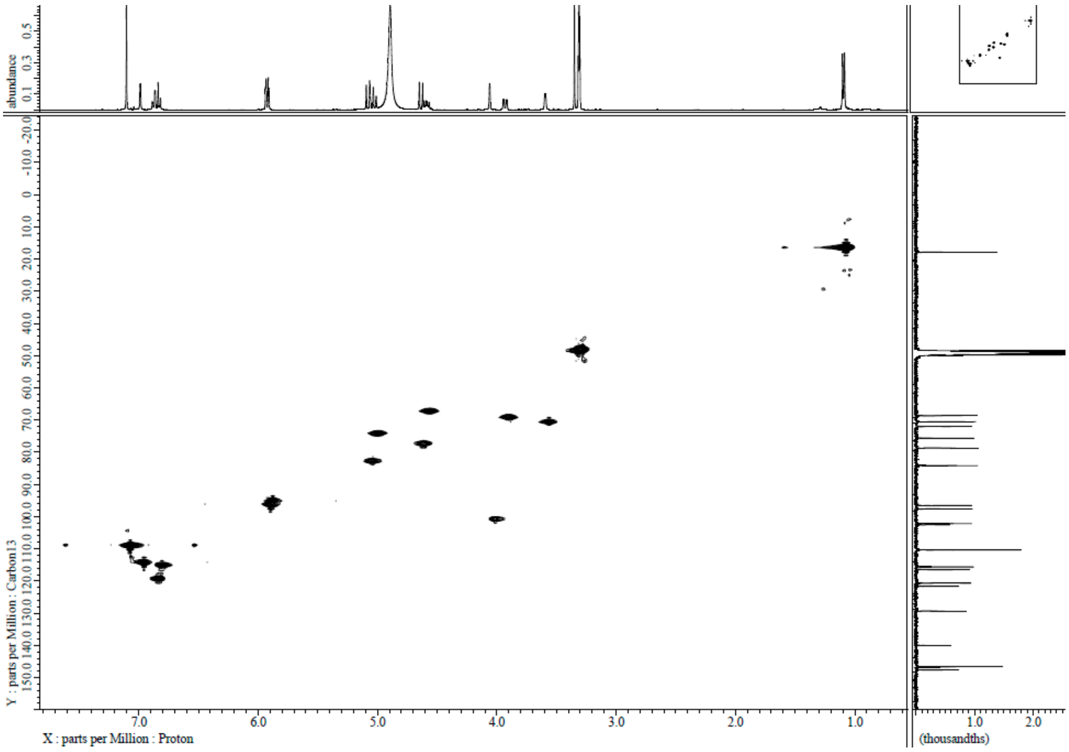

Figure S7. HSQC spectrum (CD_3OD, 400 MHz) of compound **1**.

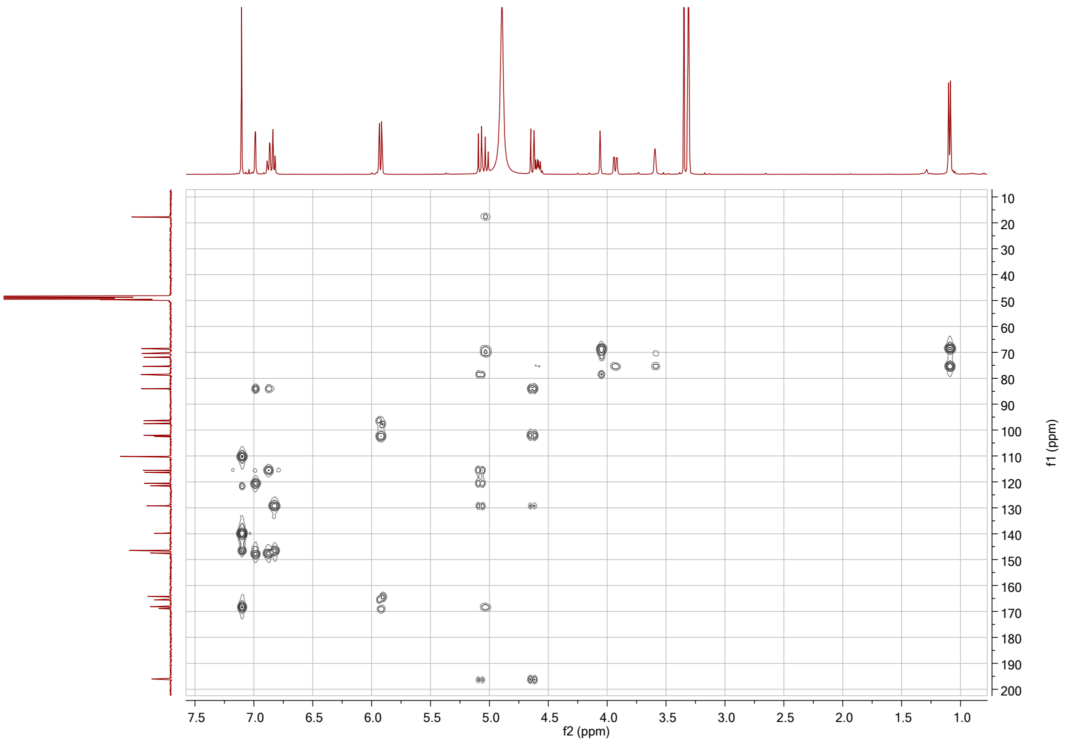

Figure S8. HMBC spectrum (CD_3OD, 400 MHz) of compound **1**.

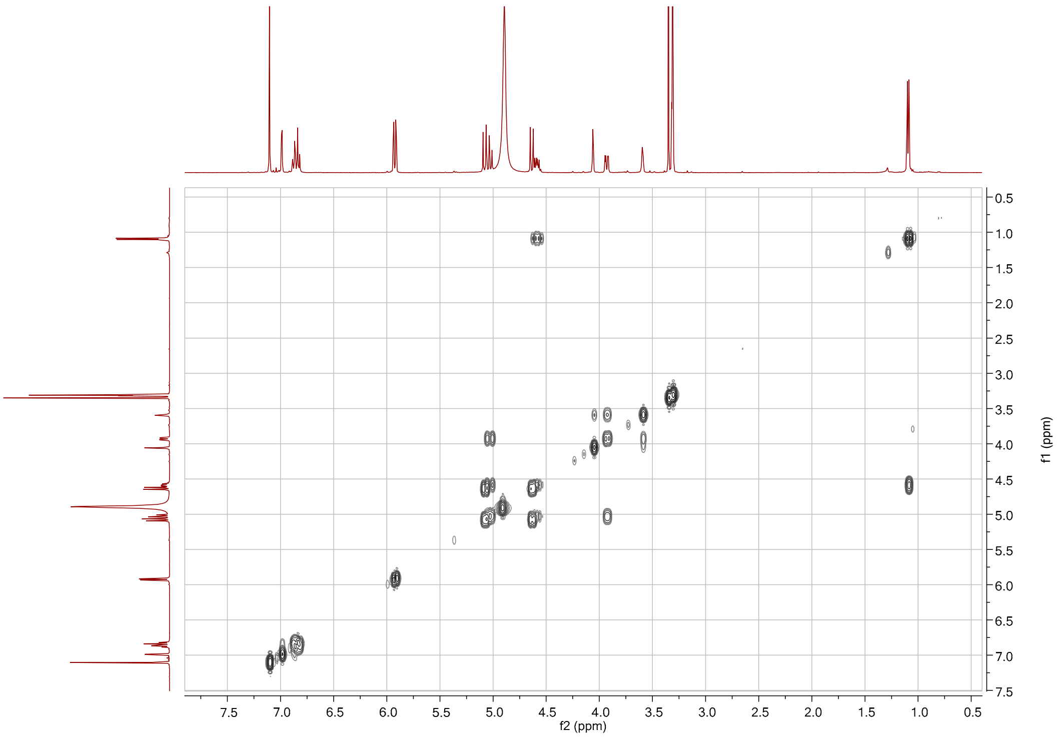

Figure S9. ^1H-^1H COSY spectrum (CD_3OD, 400 MHz) of compound **1**.

Single Mass Analysis
Tolerance = 5.0 mDa / DBE: min = -1.5, max = 50.0
Element prediction: Off
Number of isotope peaks used for i-FIT = 3
Monoisotopic Mass, Even Electron Ions
174 formula(e) evaluated with 2 results within limits (up to 50 best isotopic matches for each mass)
Elements Used:

Mass	Calc. Mass	mDa	PPM	DBE	Formula	i-FIT	i-FIT Norm	Fit Conf %	C	H	O
601.1186	601.1193	-0.7	-1.2	16.5	C28 H25 O15	450.0	0.001	99.86	28	25	15
	601.1229	-4.3	-7.2	38.5	C46 H17 O2	456.6	6.608	0.14	46	17	2

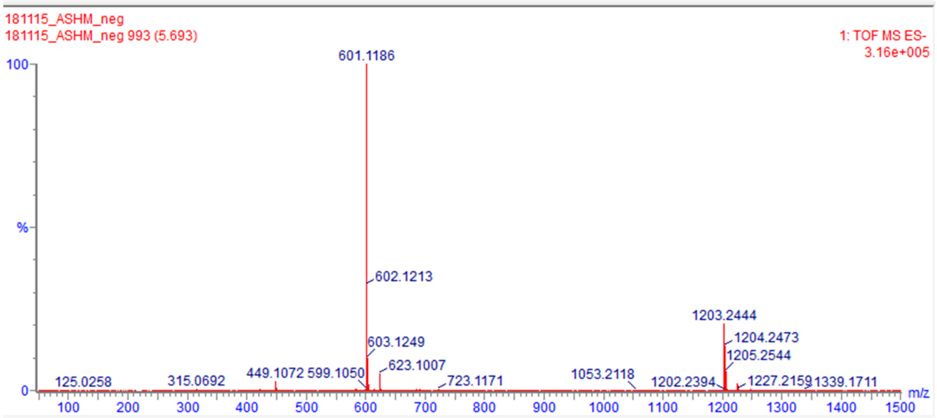

Figure S10. HR-ESI-MS of compound **2**.

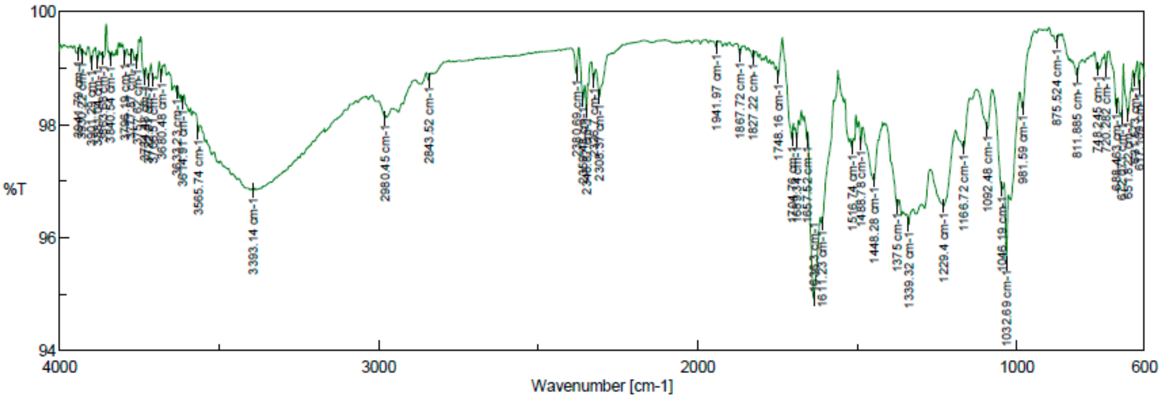

[Comment]
Sample Name ASHM8-4
Comment
User
Division
Company 공동기기실

[Data Information]
Creation Date 2019-02-13 오후 3:58
Data array type Linear data array
Horizontal Wavenumber [cm-1]
Vertical %T
Start 599.753 cm-1
End 4000.6 cm-1
Data pitch 0.964233 cm-1
Data points 3528

[Measurement Information]
Model Name FT/IR-4200typeA
Serial Number B038361018

Light Source Standard
Detector TGS
Accumulation Auto (23)
Resolution 4 cm-1
Zero Filling On
Apodization Cosine
Gain Auto (2)
Aperture Auto (7.1 mm)
Scanning Speed Auto (2 mm/sec)
Filter Auto (30000 Hz)

Figure S11. IR spectrum of compound **2**.

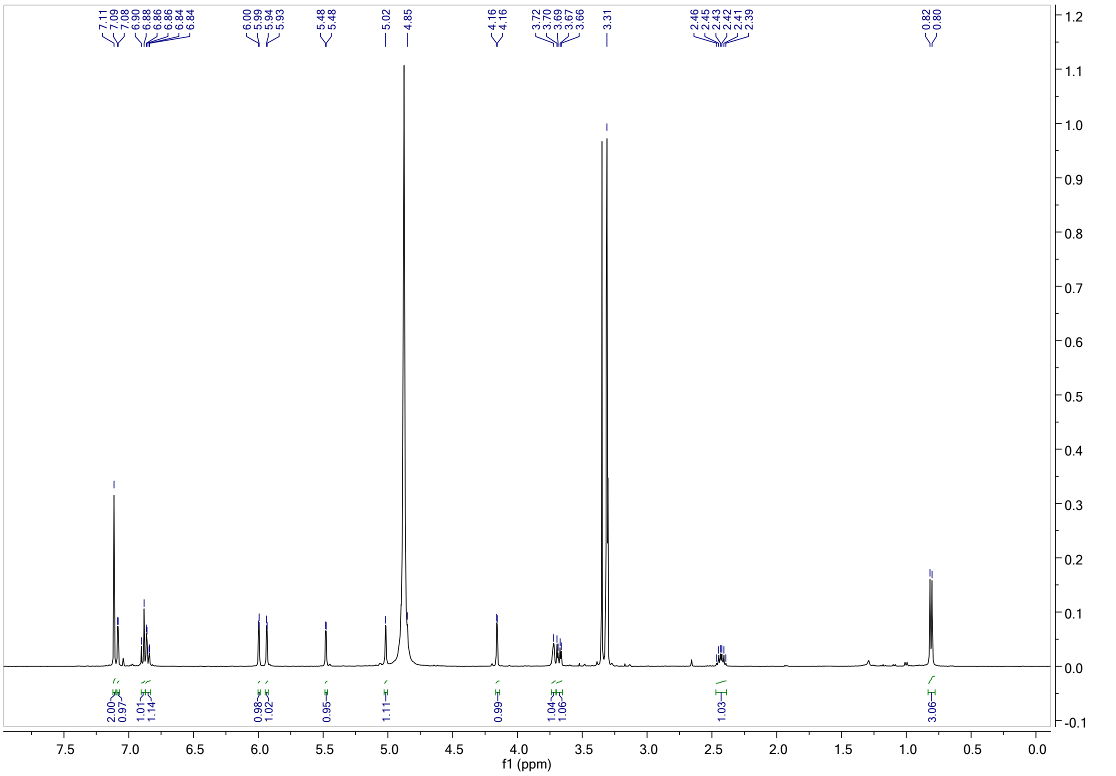

Figure S12. ^{1}H NMR spectrum (CD_3OD, 400 MHz) of compound **2**.

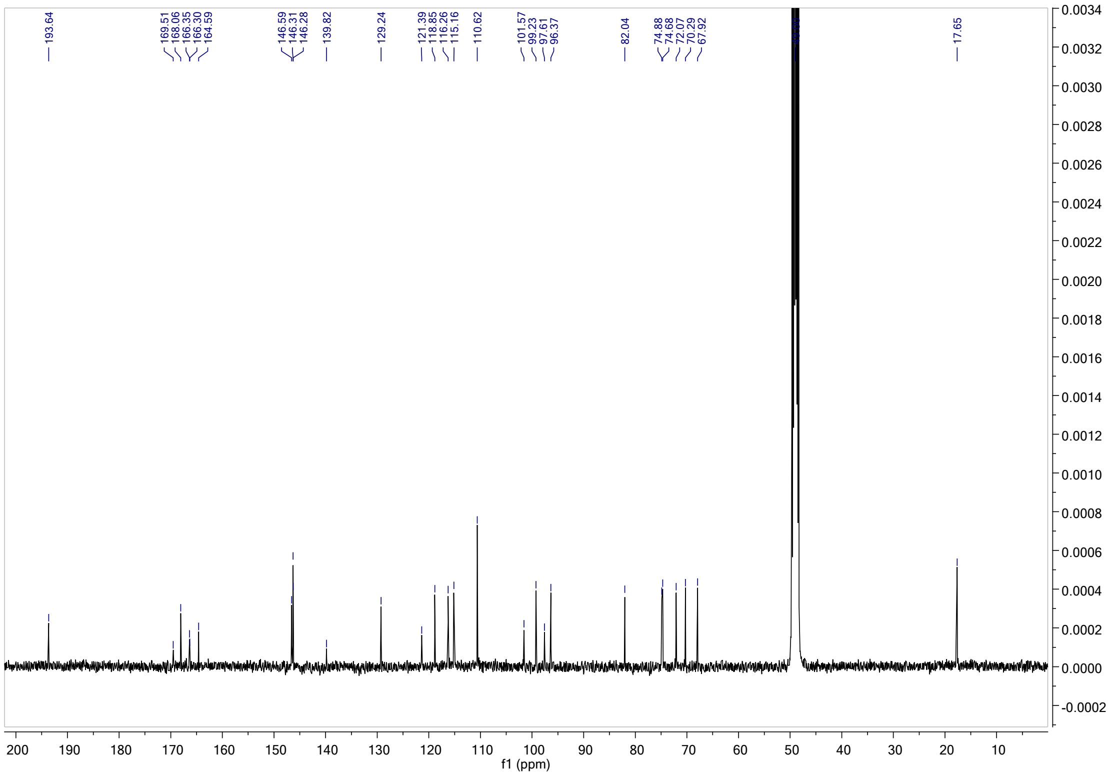

Figure S13. ^{13}C NMR spectrum (CD_3OD, 100 MHz) of compound **2**.

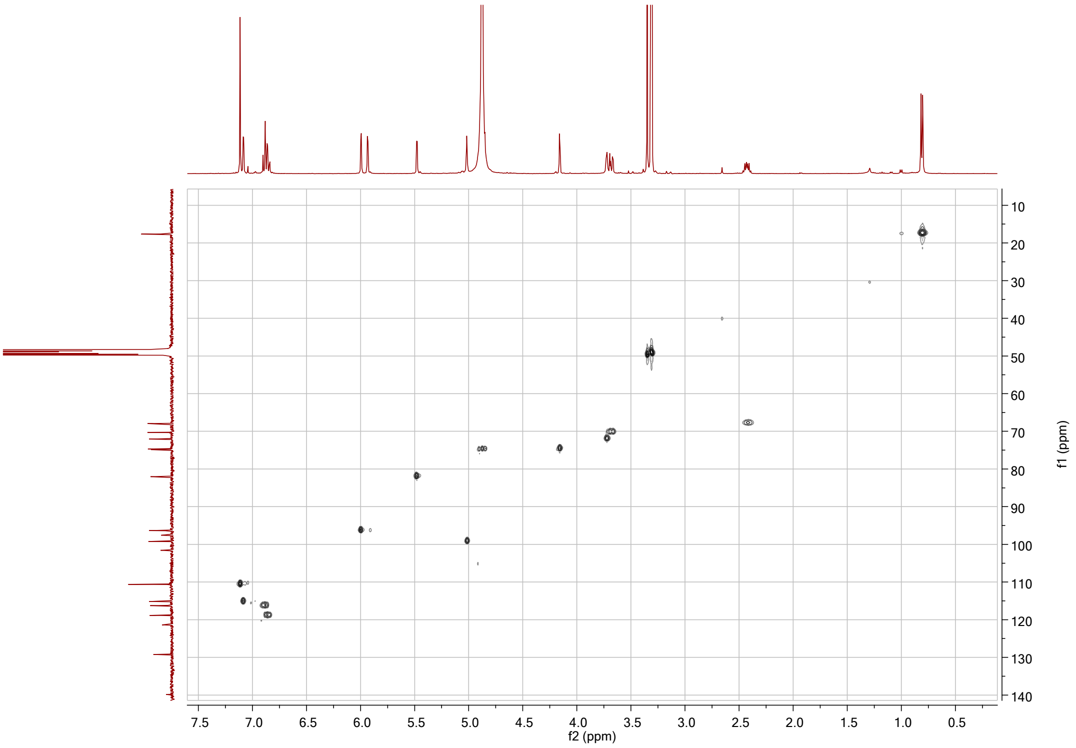

Figure S14. HSQC spectrum (CD3OD, 400 MHz) of compound **2**.

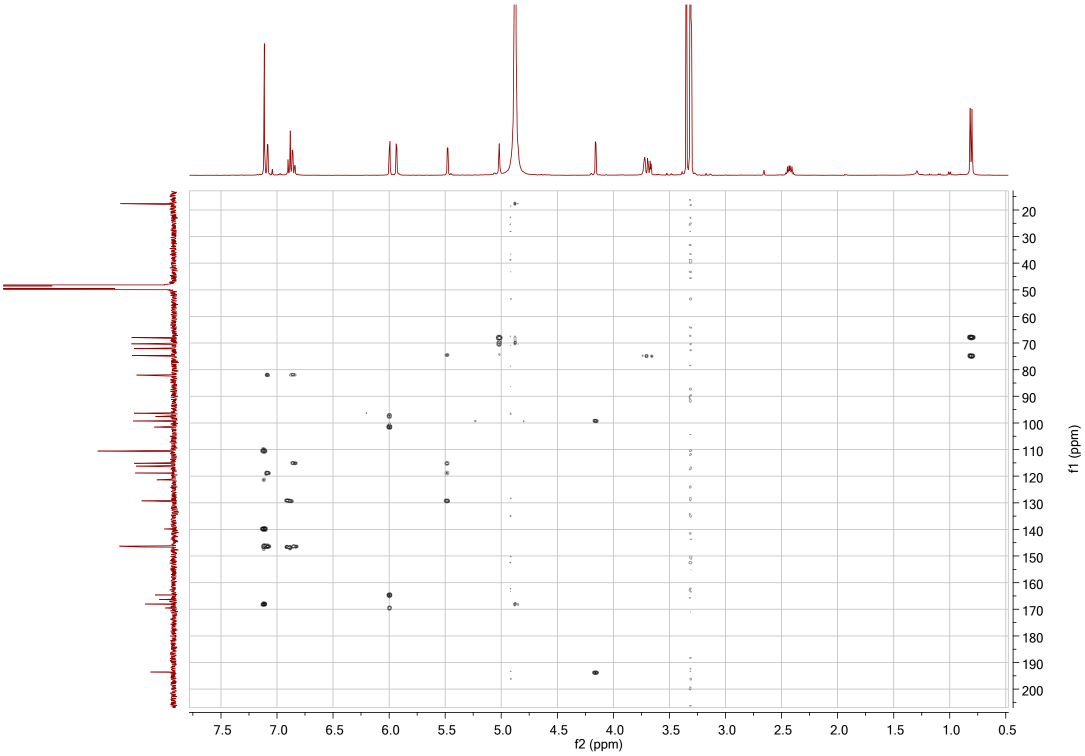

Figure S15. HMBC spectrum (CD3OD, 400 MHz) of compound **2**.

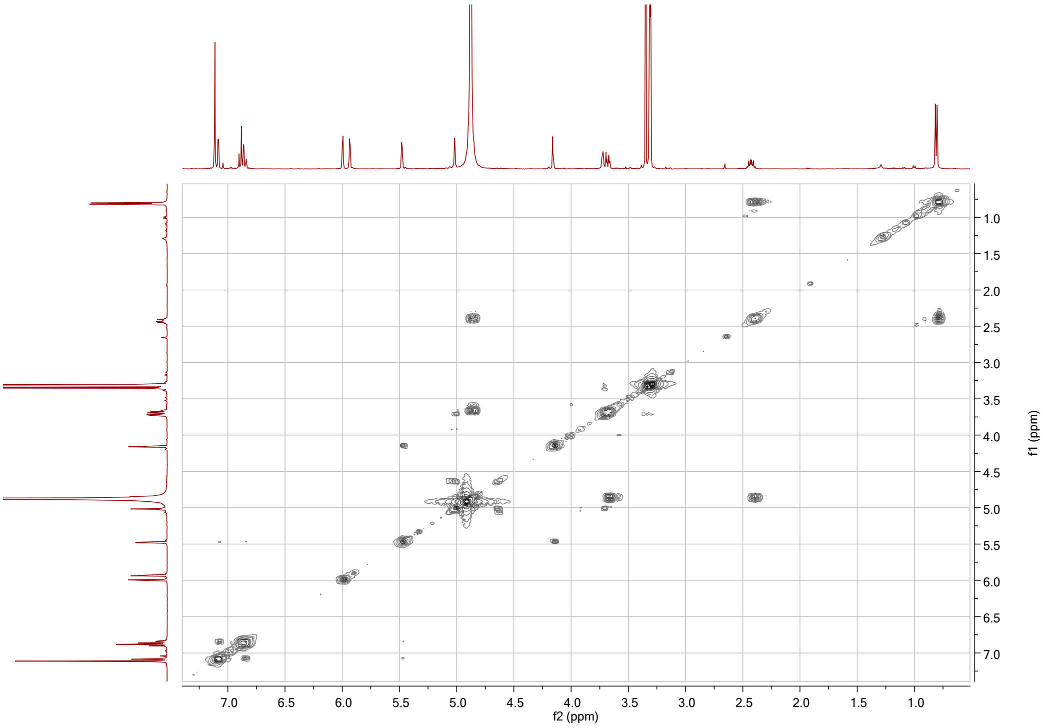

Figure S16. ^{1}H-^{1}H COSY spectrum (CD_3OD, 400 MHz) of compound **2**.

Single Mass Analysis
Tolerance = 5.0 mDa / DBE: min = -1.5, max = 50.0
Element prediction: Off
Number of isotope peaks used for i-FIT = 3
Monoisotopic Mass, Even Electron Ions
166 formula(e) evaluated with 3 results within limits (up to 50 best isotopic matches for each mass)
Elements Used:

Mass	Calc. Mass	mDa	PPM	DBE	Formula	i-FIT	i-FIT Norm	Fit Conf %	C	H	O
585.1233	585.1244	-1.1	-1.9	16.5	C28 H25 O14	323.4	0.012	98.76	28	25	14
	585.1186	4.7	8.0	25.5	C35 H21 O9	327.8	4.412	1.21	35	21	9
	585.1279	-4.6	-7.9	38.5	C46 H17 O	331.6	8.165	0.03	46	17	1

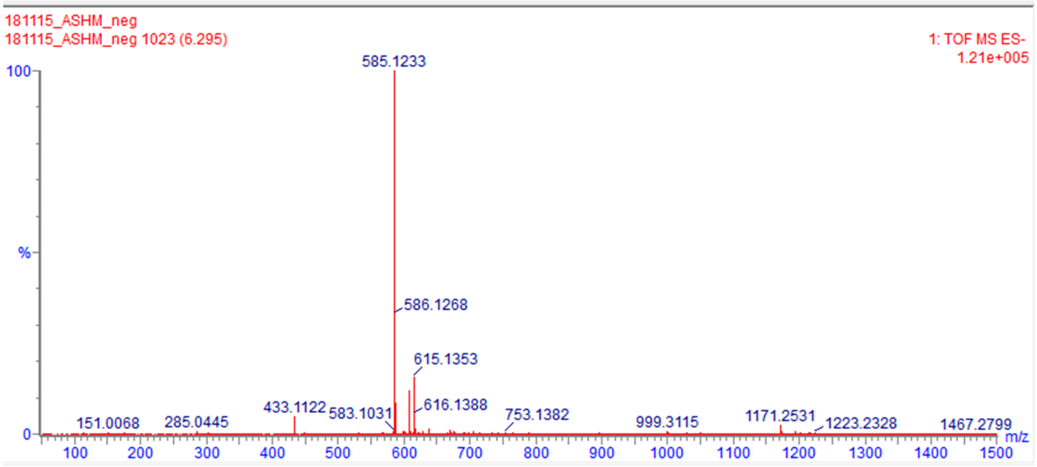

Figure S17. HR-ESI-MS of compound **3**.

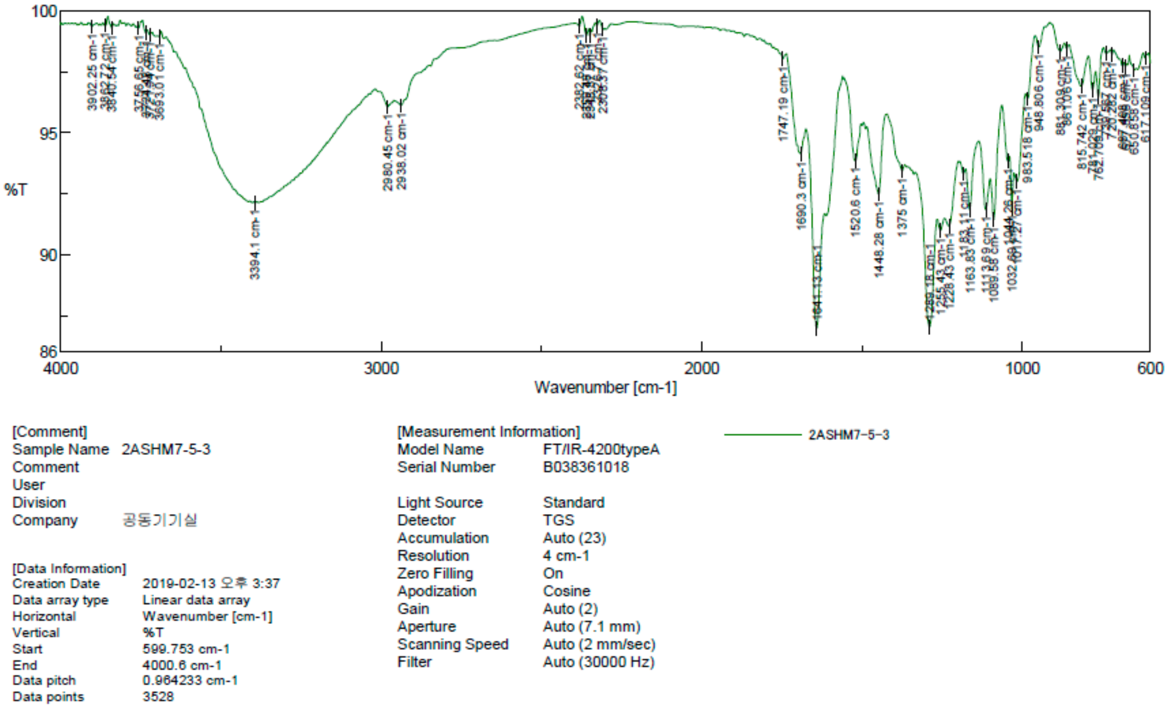

Figure S18. IR spectrum of compound **3**.

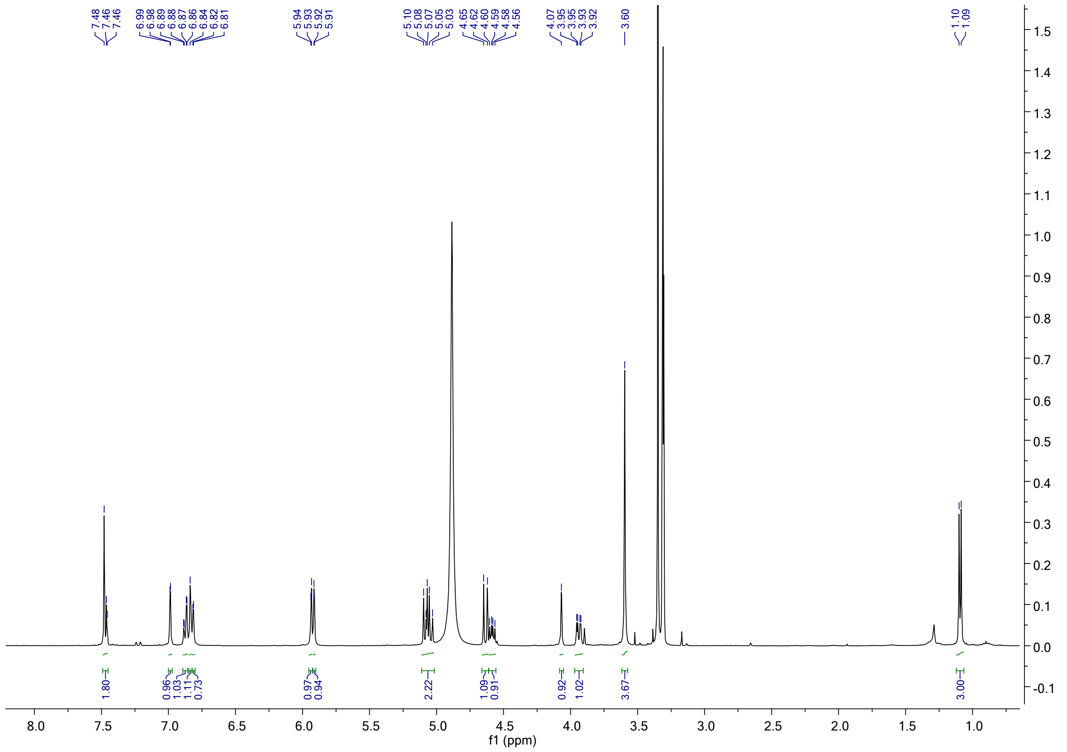

Figure S19. ^{1}H NMR spectrum (CD_3OD, 400 MHz) of compound **3**.

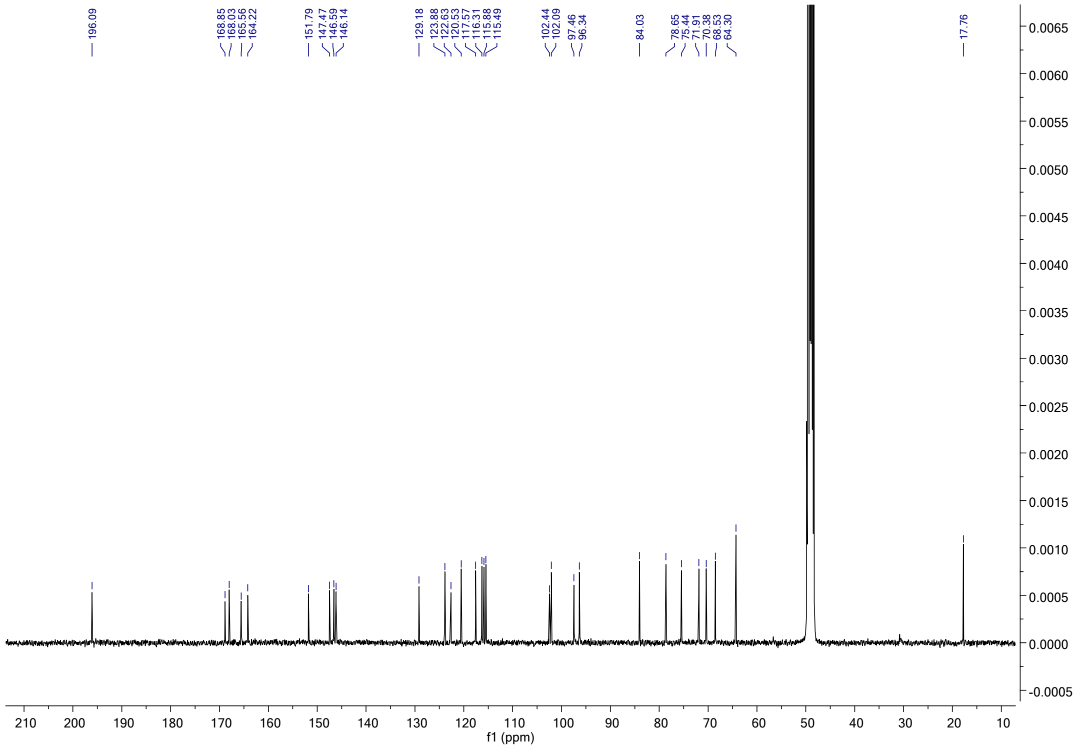

Figure S20. ^{13}C NMR spectrum (CD_3OD, 100 MHz) of compound **3**.

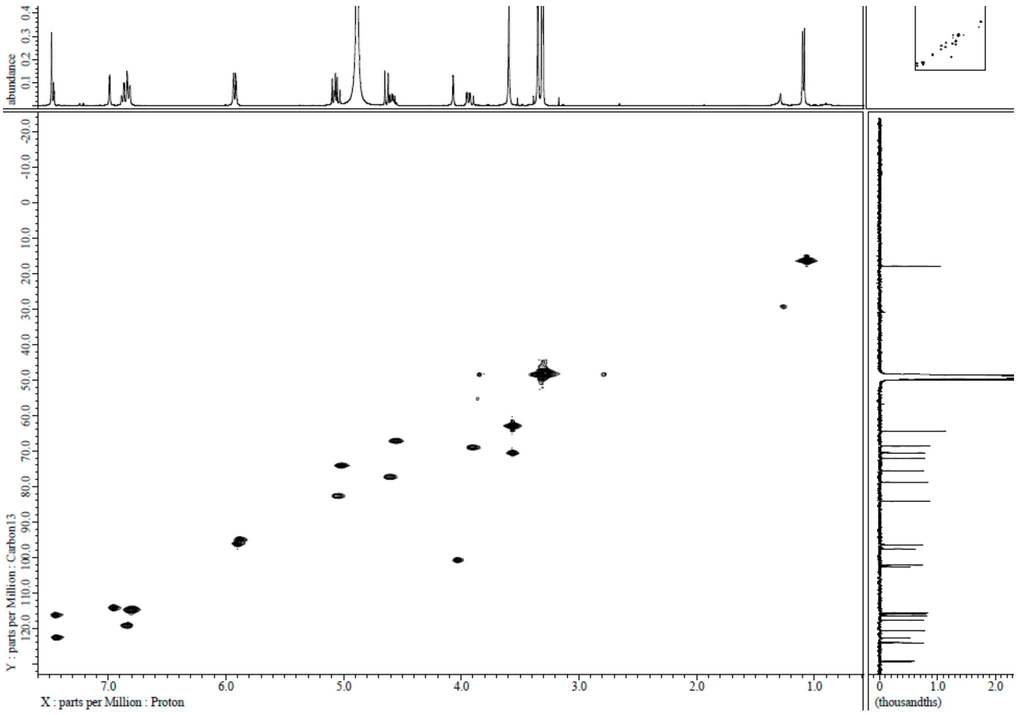

Figure S21. HSQC spectrum (CD_3OD, 400 MHz) of compound **3**.

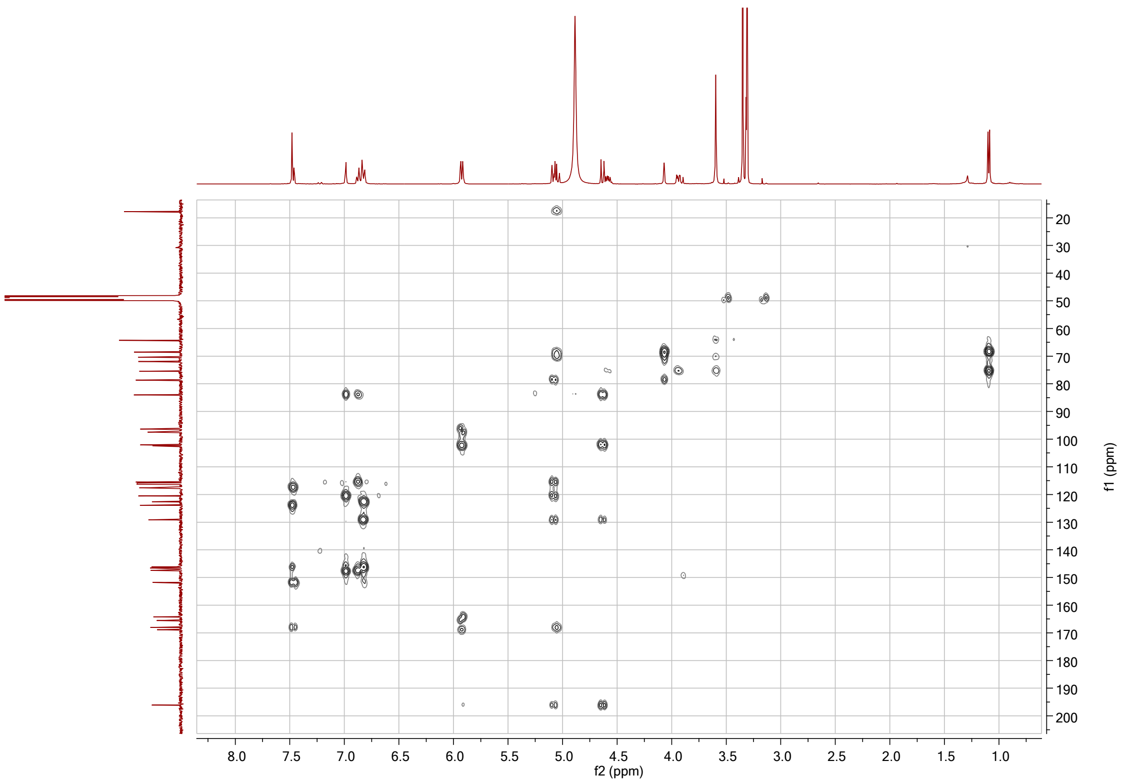

Figure S22. HMBC spectrum (CD_3OD, 400 MHz) of compound **3**.

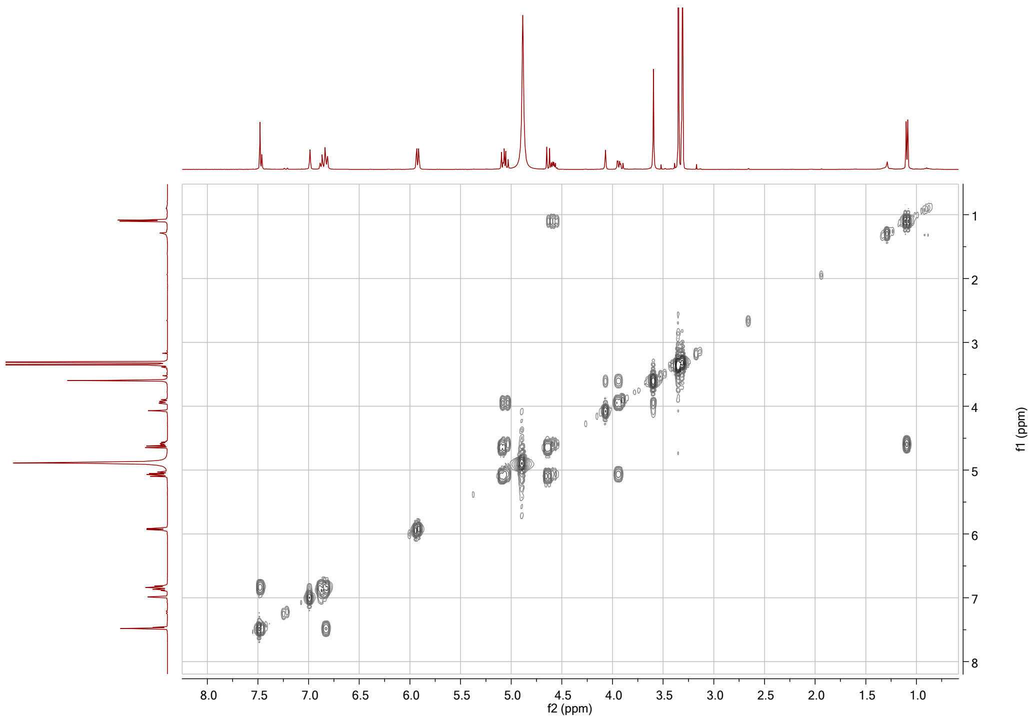

Figure S23. ^1H-^1H COSY spectrum (CD_3OD, 400 MHz) of compound **3**.

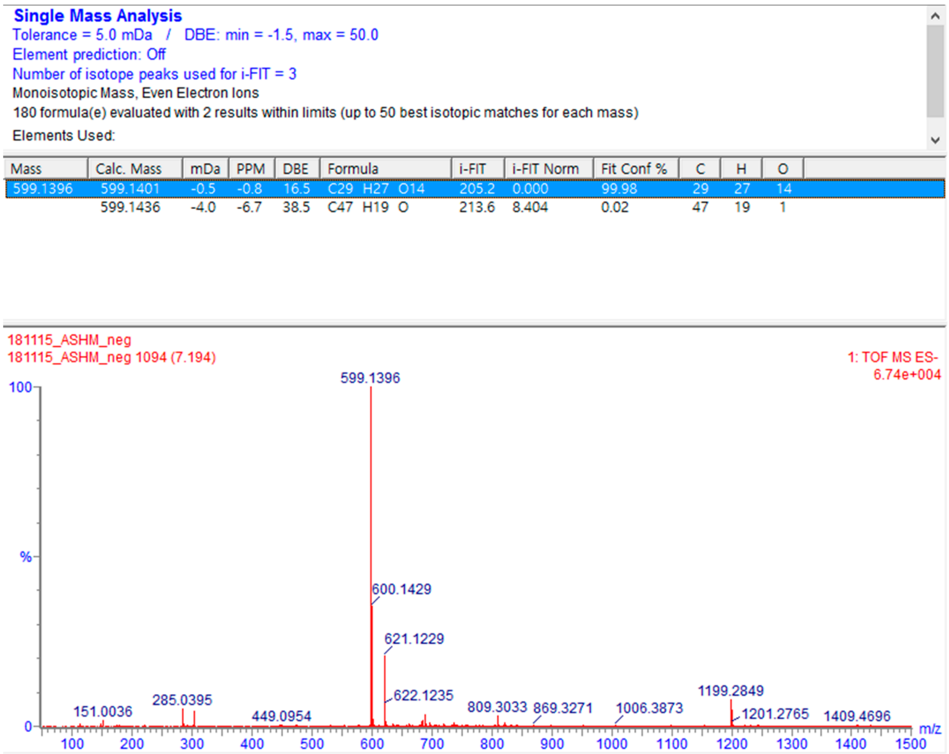

Figure S24. HR-ESI-MS of compound **4**.

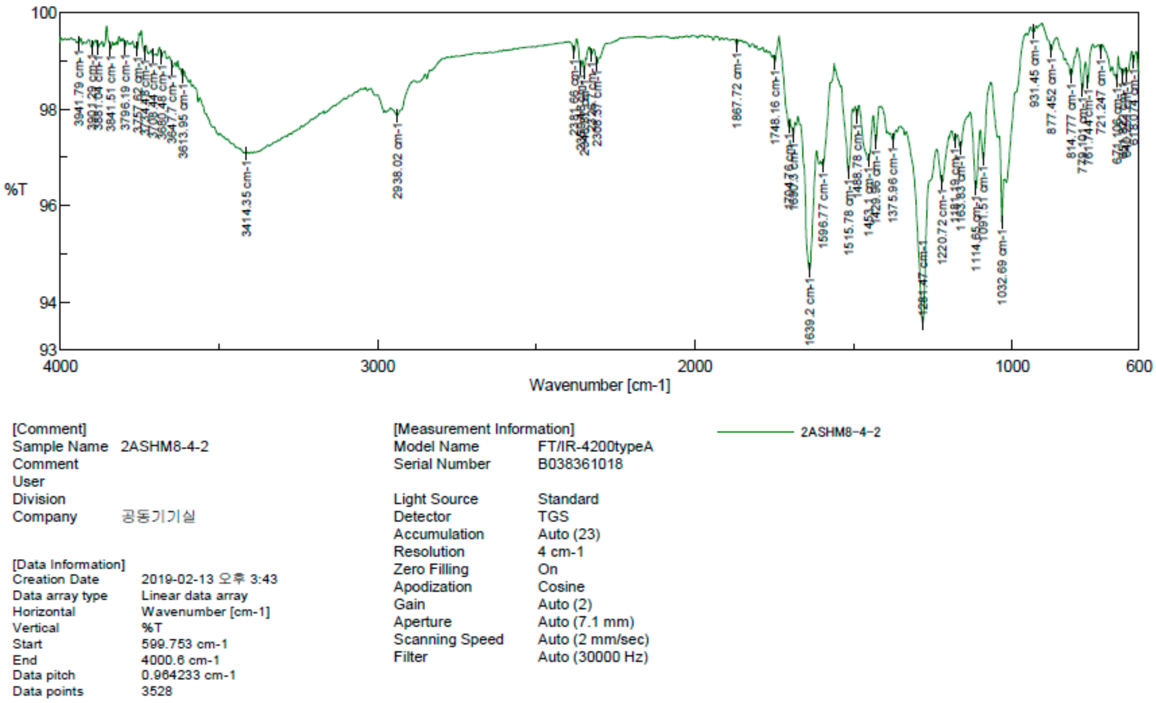

Figure S25. IR spectrum of compound **4**.

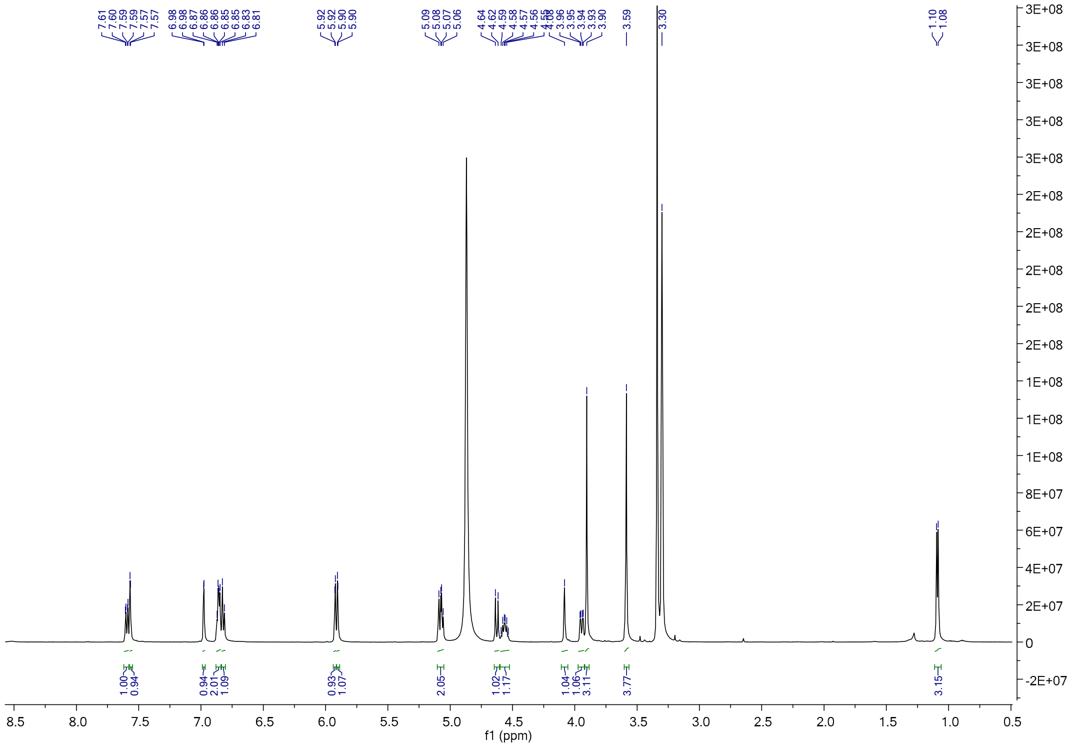

Figure S26. ^{1}H NMR spectrum (CD_3OD, 500 MHz) of compound **4**.

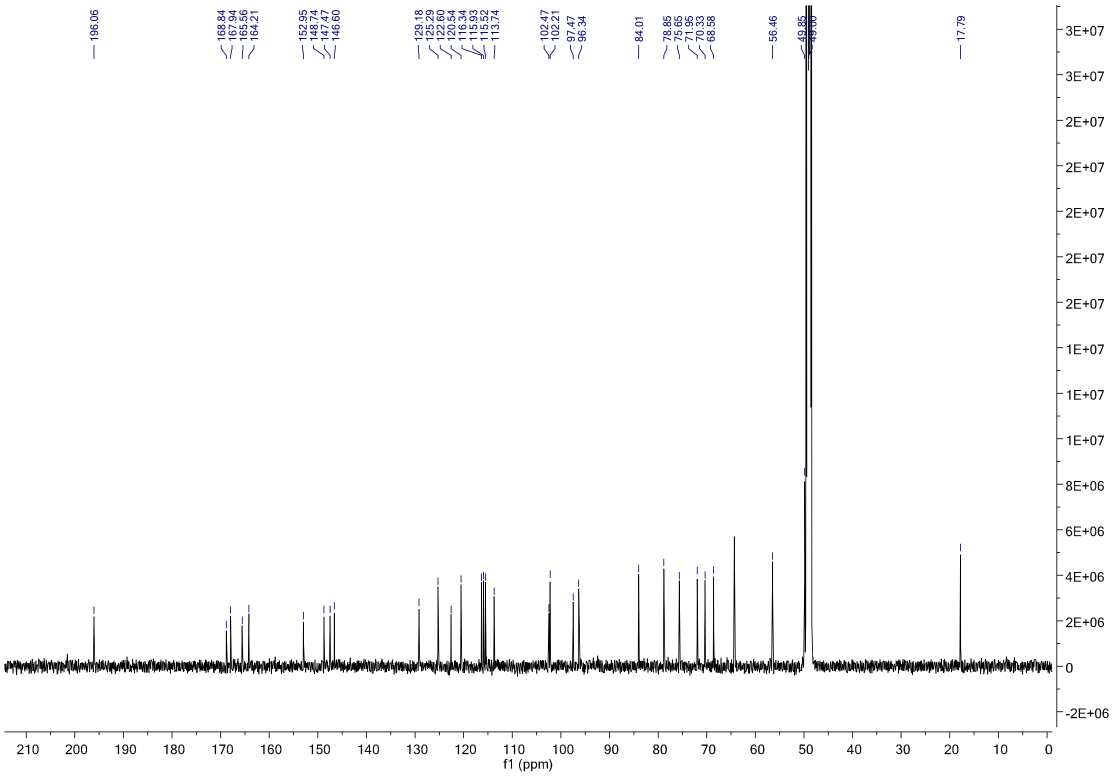

Figure S27. ^{13}C NMR spectrum (CD_3OD, 125 MHz) of compound **4**.

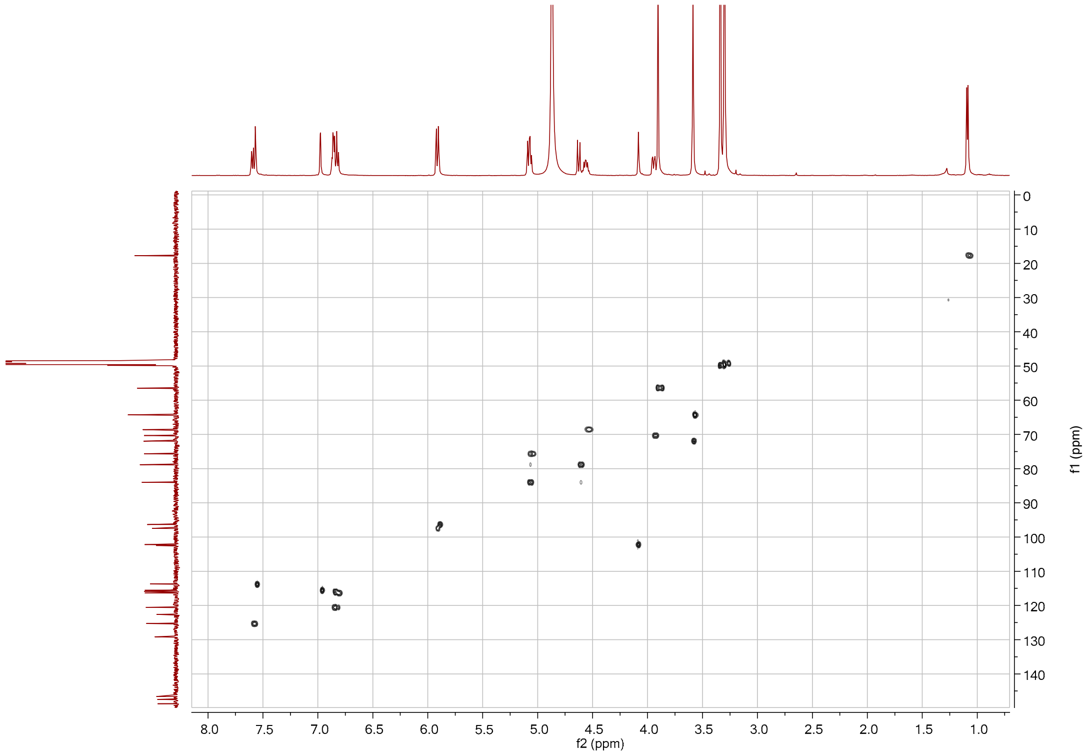

Figure S28. HSQC spectrum (CD3OD, 500 MHz) of compound **4**.

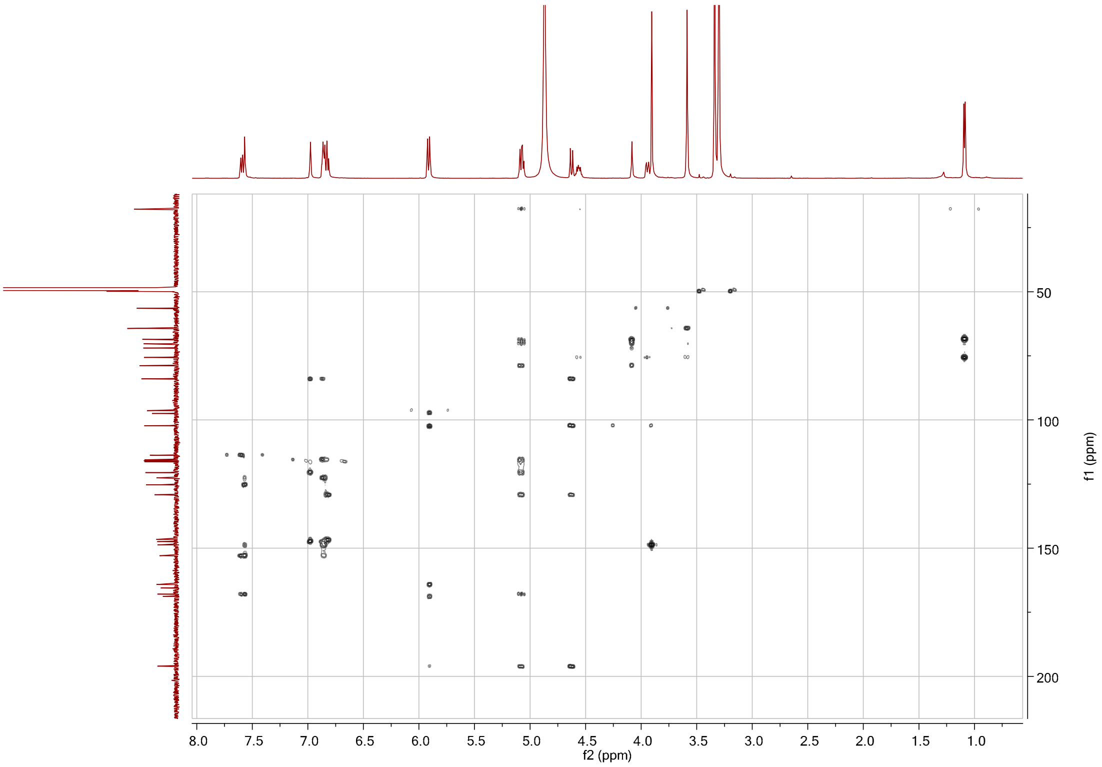

Figure S29. HMBC spectrum (CD3OD, 500 MHz) of compound **4**.

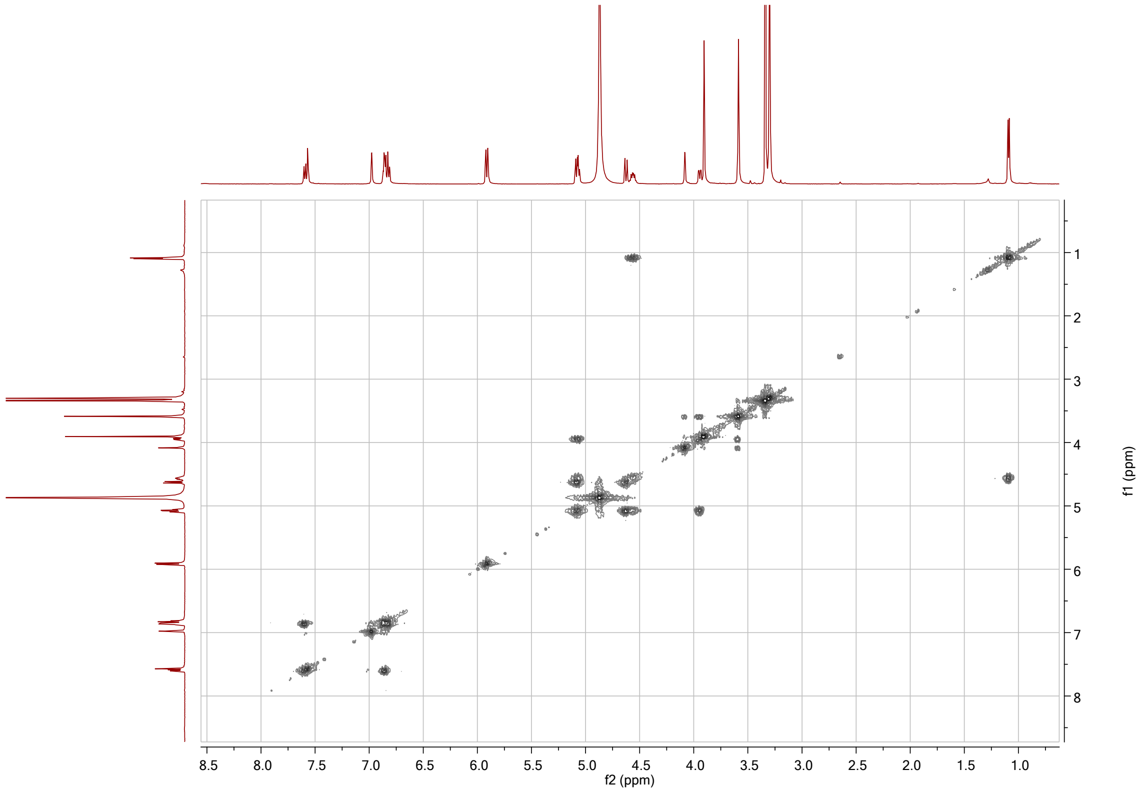

Figure S30. ^1H-^1H COSY spectrum (CD_3OD, 500 MHz) of compound **4**.

Single Mass Analysis
Tolerance = 5.0 mDa / DBE: min = -1.5, max = 50.0
Element prediction: Off
Number of isotope peaks used for i-FIT = 3
Monoisotopic Mass, Even Electron Ions
180 formula(e) evaluated with 2 results within limits (up to 50 best isotopic matches for each mass)
Elements Used:

Mass	Calc. Mass	mDa	PPM	DBE	Formula	i-FIT	i-FIT Norm	Fit Conf %	C	H	O
599.1382	599.1401	-1.9	-3.2	16.5	C29 H27 O14	176.4	0.255	77.49	29	27	14
	599.1342	4.0	6.7	25.5	C36 H23 O9	177.6	1.491	22.51	36	23	9

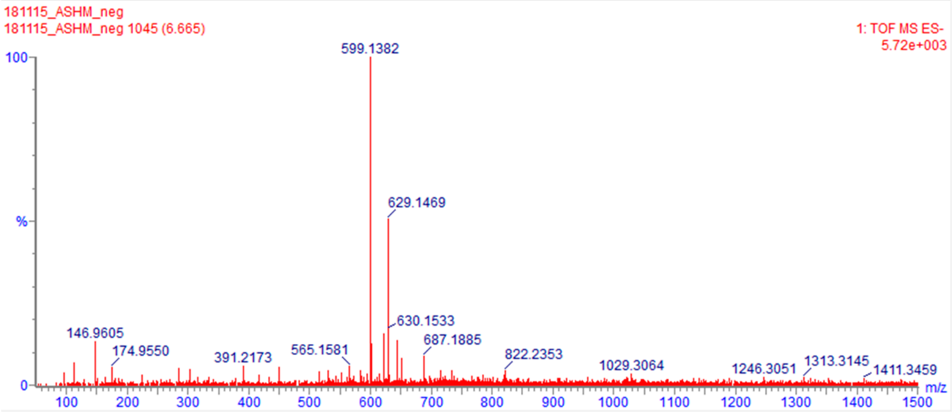

Figure S31. HR-ESI-MS of compound **5**.

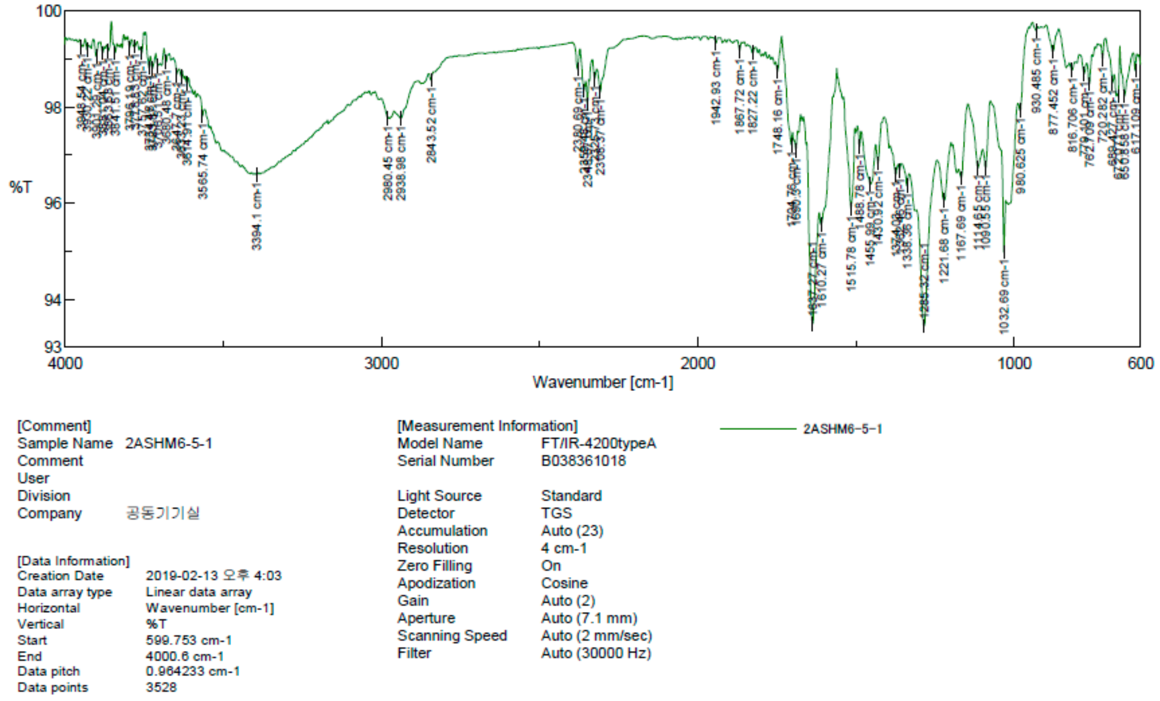

Figure S32. IR spectrum of compound **5**.

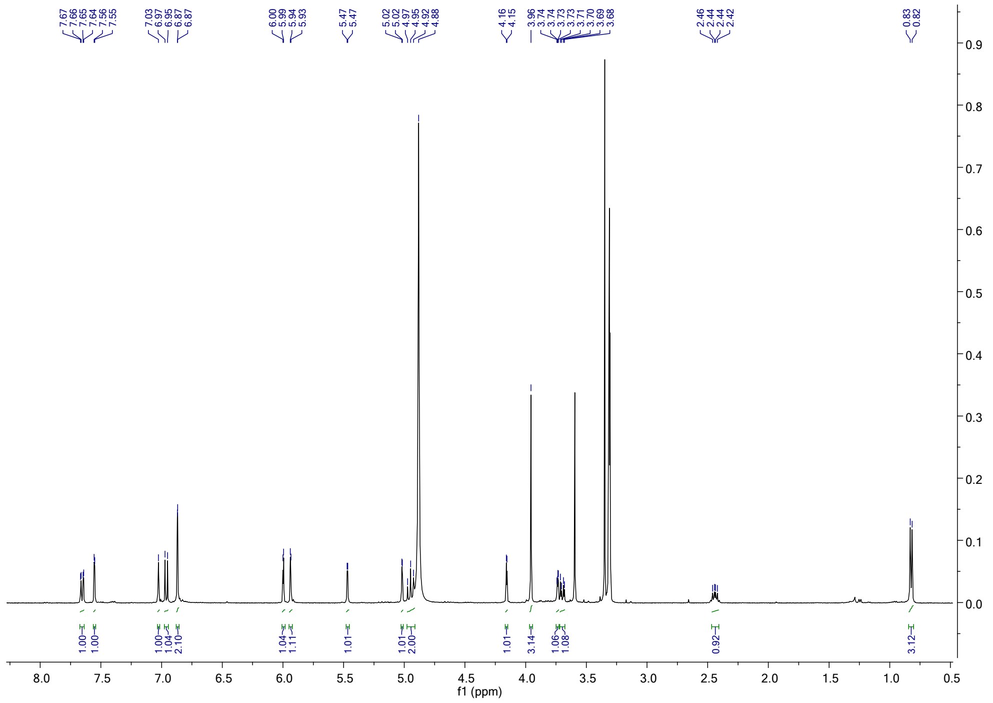

Figure S33. ^{1}H NMR spectrum (CD_3OD, 400 MHz) of compound **5**.

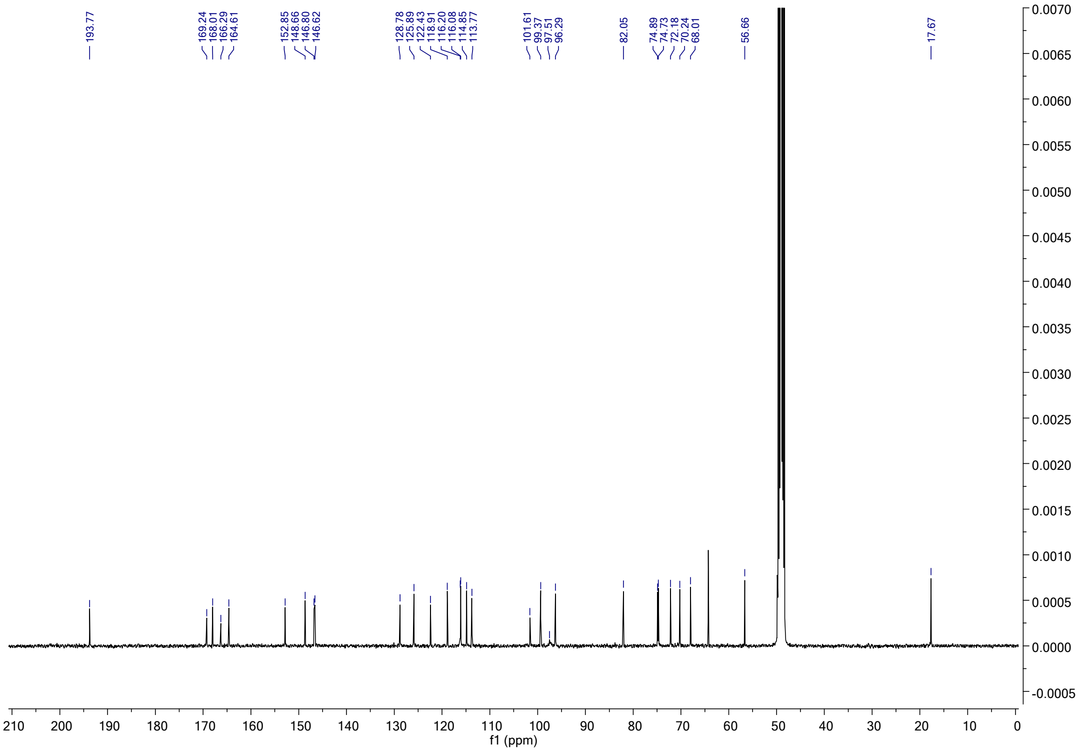

Figure S34. ^{13}C NMR spectrum (CD_3OD, 100 MHz) of compound **5**.

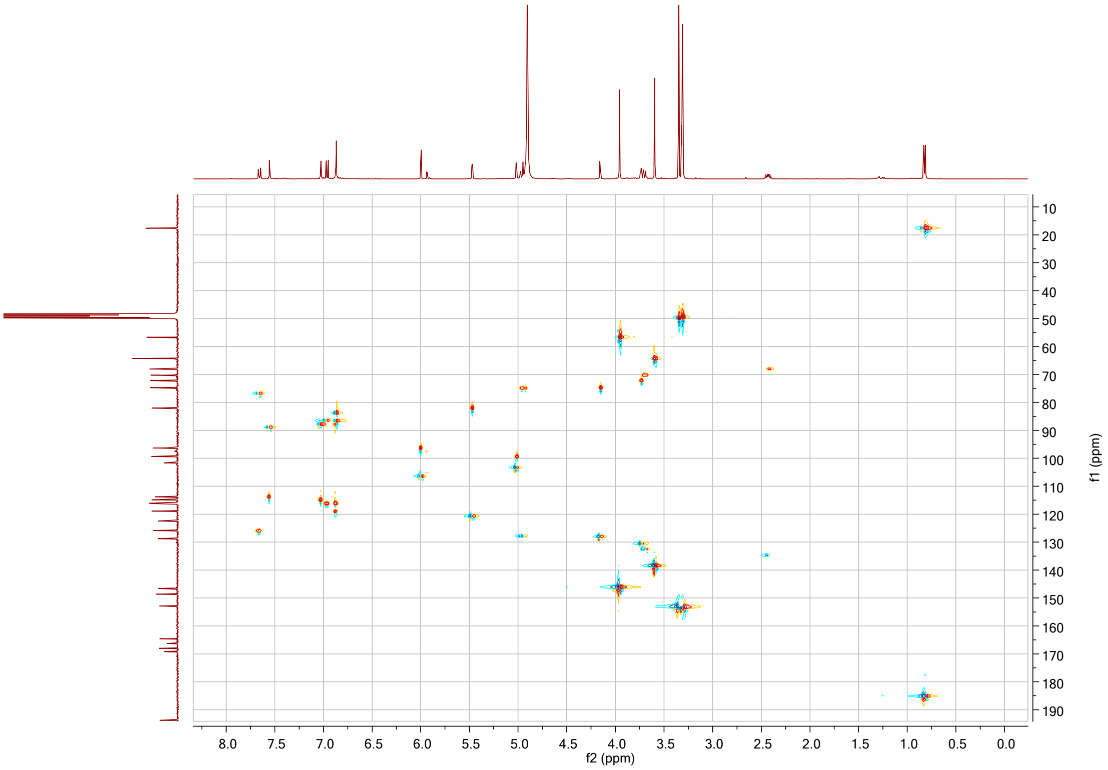

Figure S35. HSQC spectrum (CD_3OD, 400 MHz) of compound **5**.

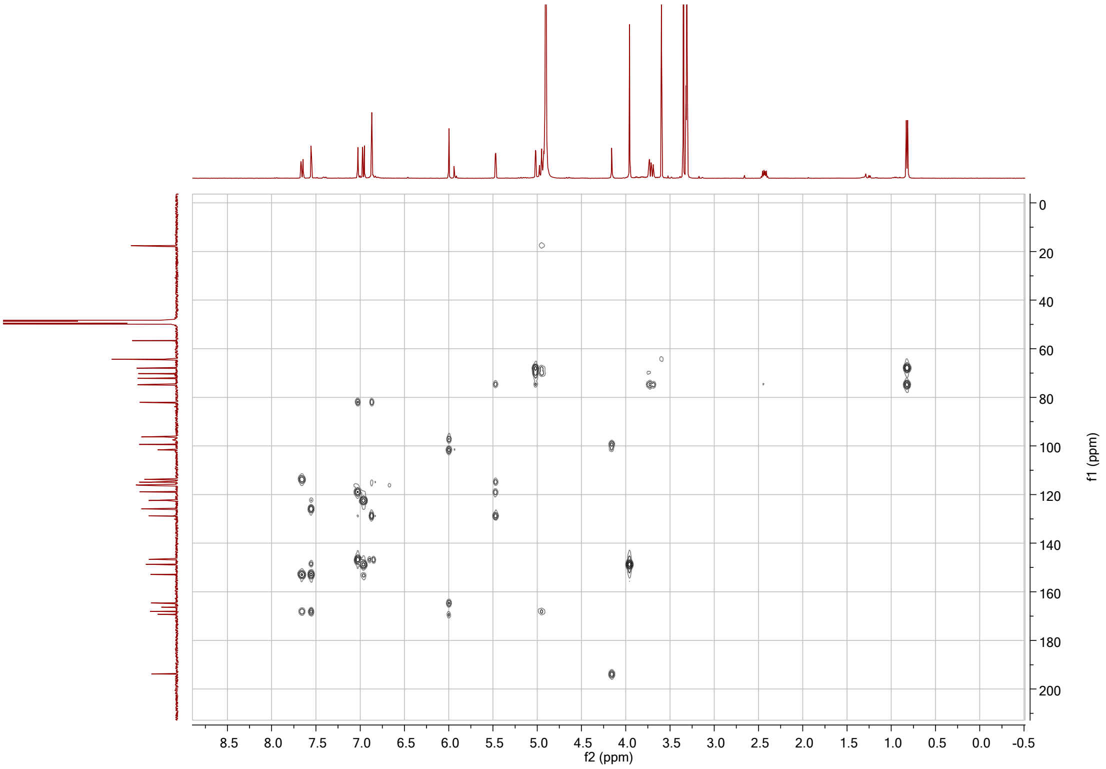

Figure S36. HMBC spectrum (CD_3OD, 400 MHz) of compound **5**.

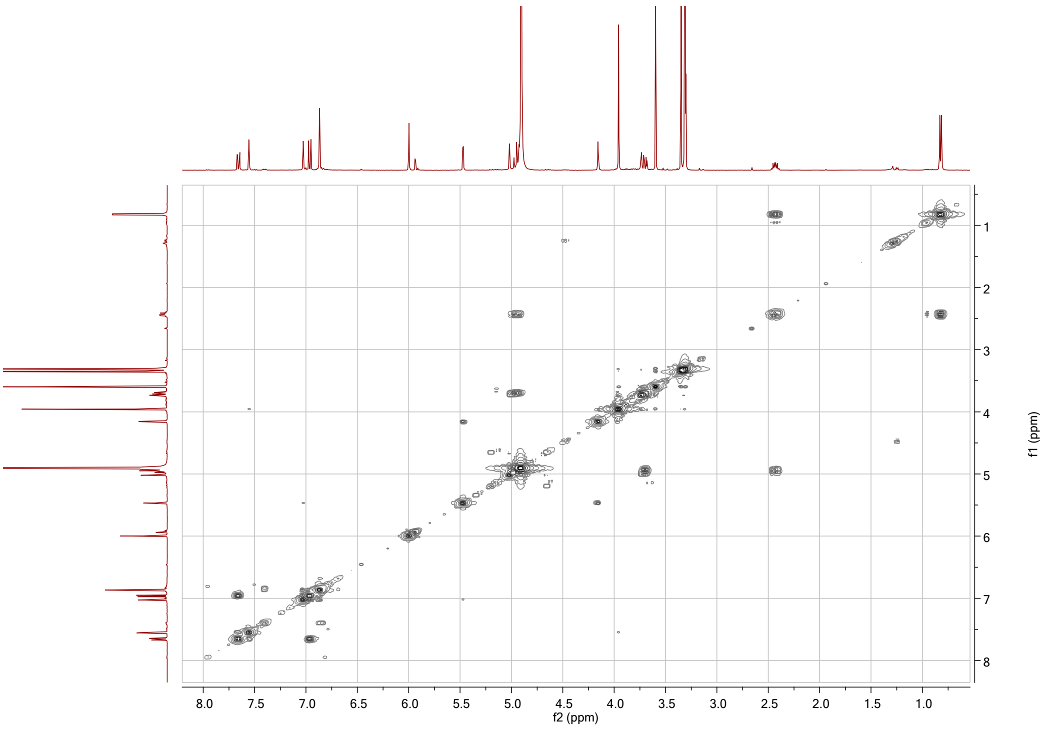

Figure S37. ^1H-^1H COSY spectrum (CD_3OD, 400 MHz) of compound **5**.

Single Mass Analysis
Tolerance = 5.0 mDa / DBE: min = -1.5, max = 50.0
Element prediction: Off
Number of isotope peaks used for i-FIT = 3
Monoisotopic Mass, Even Electron Ions
158 formula(e) evaluated with 2 results within limits (up to 50 best isotopic matches for each mass)
Elements Used:

Mass	Calc. Mass	mDa	PPM	DBE	Formula	i-FIT	i-FIT Norm	Fit Conf %	C	H	O
569.1301	569.1295	0.6	1.1	16.5	C28 H25 O13	78.3	0.000	100.00	28	25	13
	569.1330	-2.9	-5.1	38.5	C46 H17	91.9	13.564	0.00	46	17	

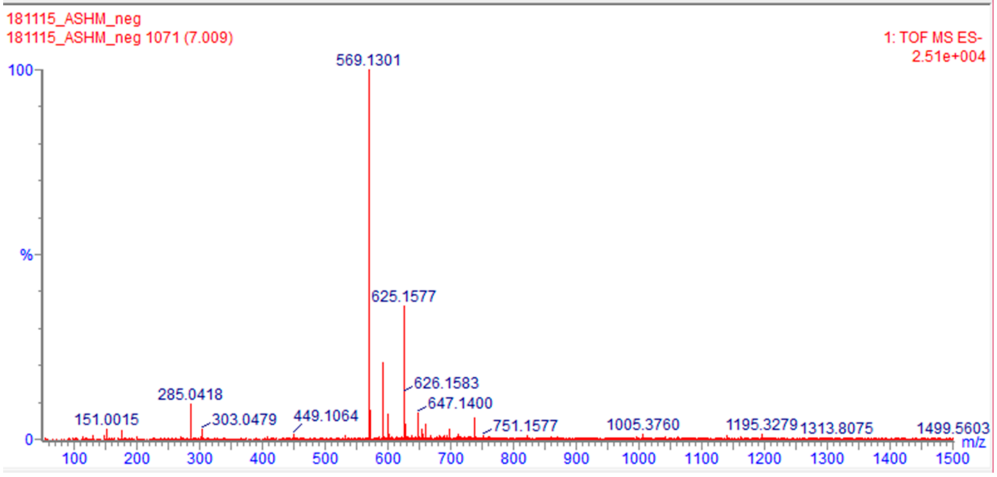

Figure S38. HR-ESI-MS of compound **6**.

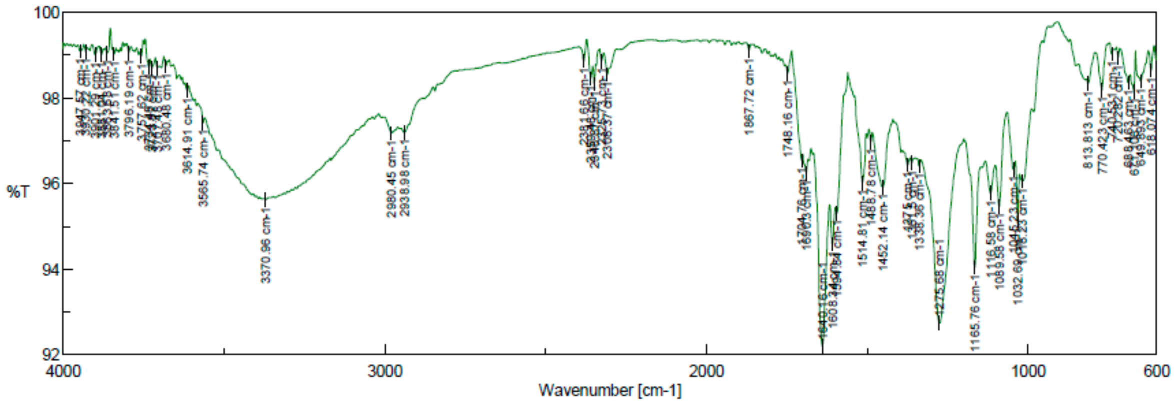

[Comment]
Sample Name 2ASHM8-4-1
Comment
User
Division
Company 공동기기실

[Data Information]
Creation Date 2019-02-13 오후 3:53
Data array type Linear data array
Horizontal Wavenumber [cm-1]
Vertical %T
Start 599.753 cm-1
End 4000.6 cm-1
Data pitch 0.964233 cm-1
Data points 3528

[Measurement Information]
Model Name FT/IR-4200typeA
Serial Number B038361018

Light Source Standard
Detector TGS
Accumulation Auto (23)
Resolution 4 cm-1
Zero Filling On
Apodization Cosine
Gain Auto (2)
Aperture Auto (7.1 mm)
Scanning Speed Auto (2 mm/sec)
Filter Auto (30000 Hz)

Figure S39. IR spectrum of compound **6**.

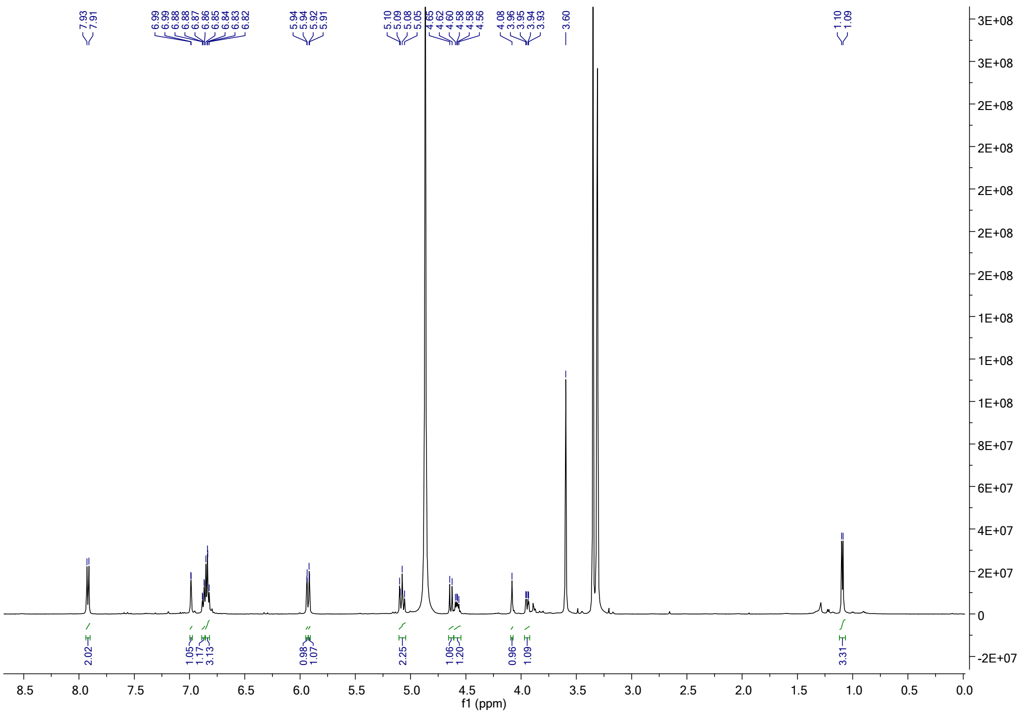

Figure S40. ^{1}H NMR spectrum (CD_3OD, 500 MHz) of compound **6**.

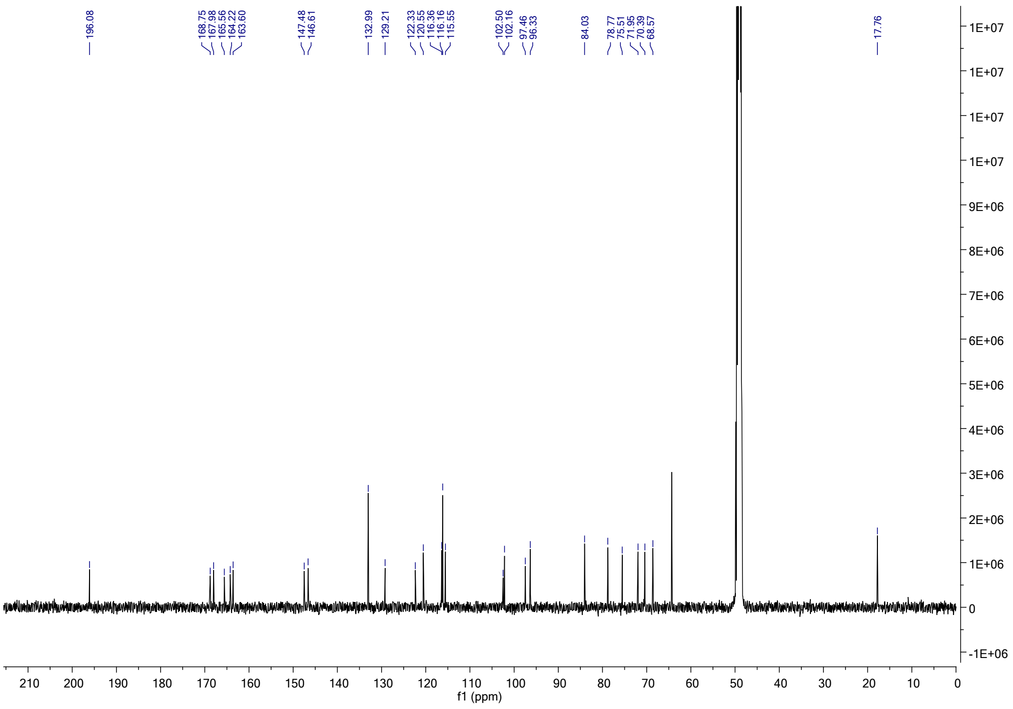

Figure S41. ^{13}C NMR spectrum (CD_3OD, 125 MHz) of compound **6**.

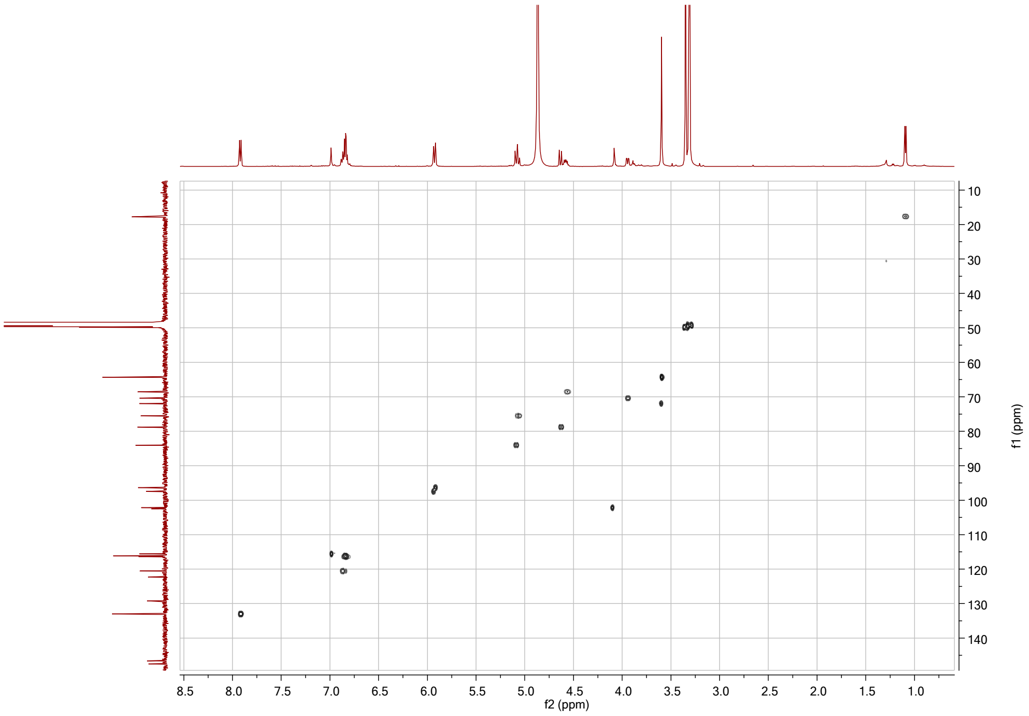

Figure S42. HSQC spectrum (CD3OD, 500 MHz) of compound **6**.

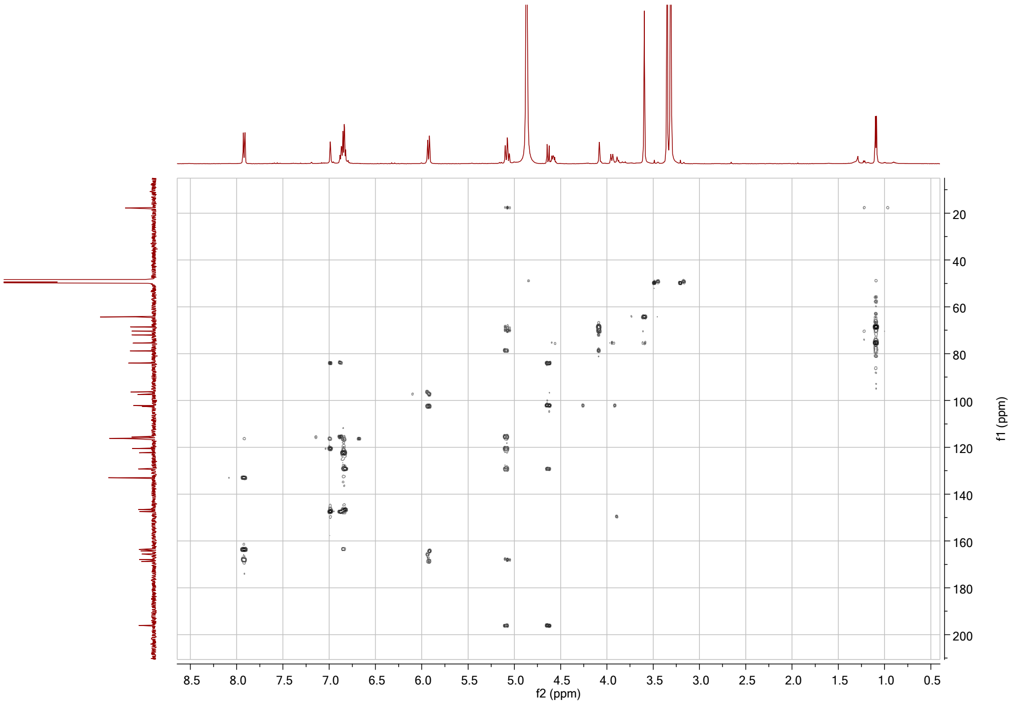

Figure S43. HMBC spectrum (CD3OD, 500 MHz) of compound **6**.

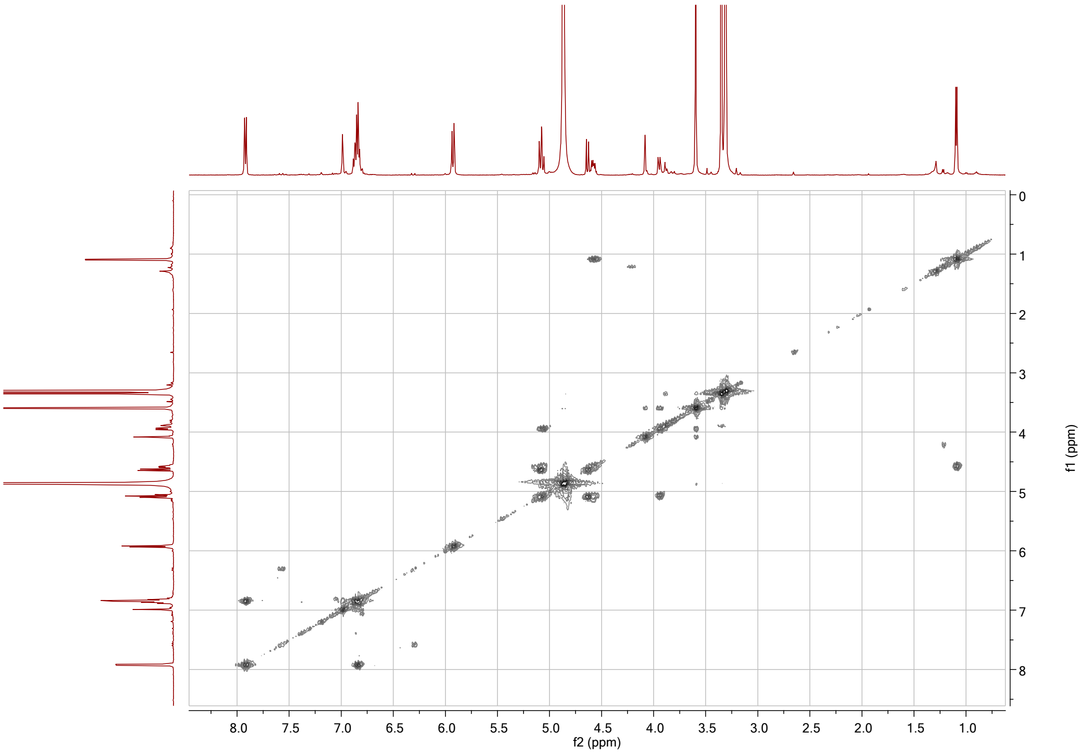

Figure S44. ^{1}H-^{1}H COSY spectrum (CD_3OD, 500 MHz) of compound **6**.

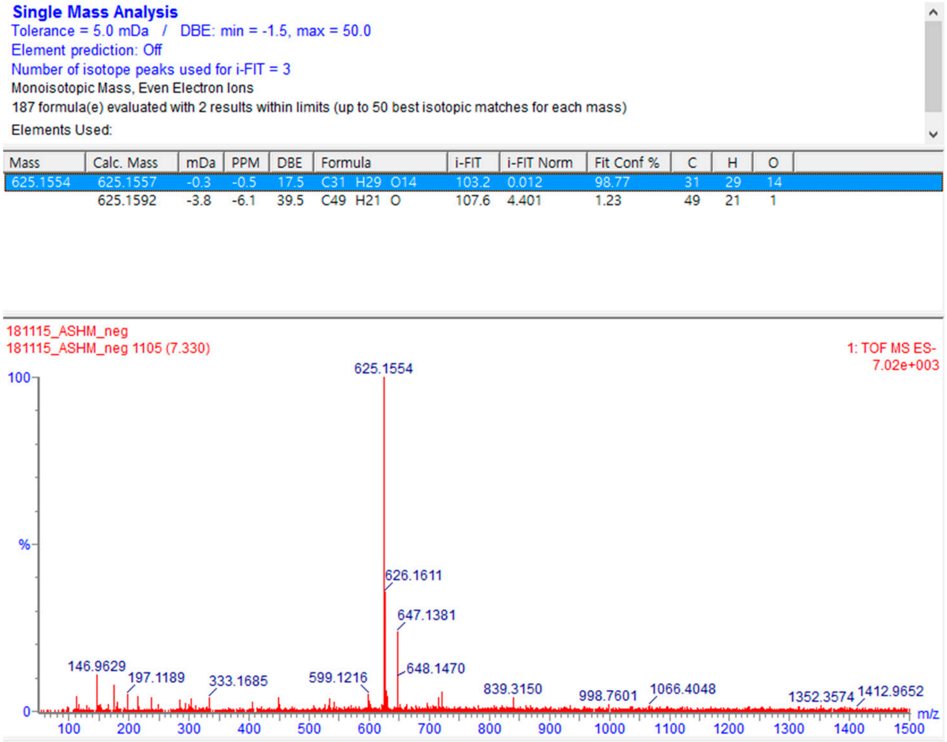

Figure S45. HR-ESI-MS of compound **7**.

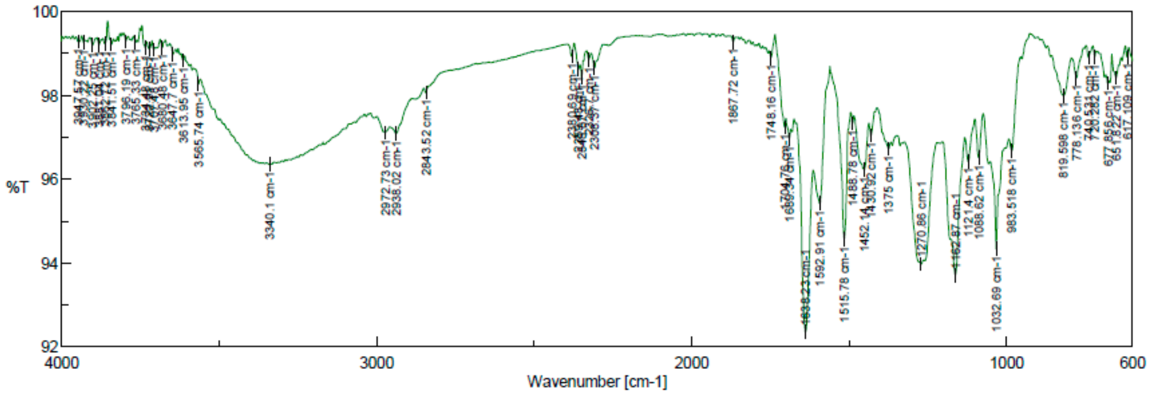

[Comment]
Sample Name 2ASHM7-4-1
Comment
User
Division
Company 공동기기실

[Data Information]
Creation Date 2019-02-13 오후 3:48
Data array type Linear data array
Horizontal Wavenumber [cm-1]
Vertical %T
Start 599.753 cm-1
End 4000.6 cm-1
Data pitch 0.964233 cm-1
Data points 3528

[Measurement Information]
Model Name FT/IR-4200typeA
Serial Number B038361018

Light Source Standard
Detector TGS
Accumulation Auto (24)
Resolution 4 cm-1
Zero Filling On
Apodization Cosine
Gain Auto (2)
Aperture Auto (7.1 mm)
Scanning Speed Auto (2 mm/sec)
Filter Auto (30000 Hz)

2ASHM7-4-1

Figure S46. IR spectrum of compound **7**.

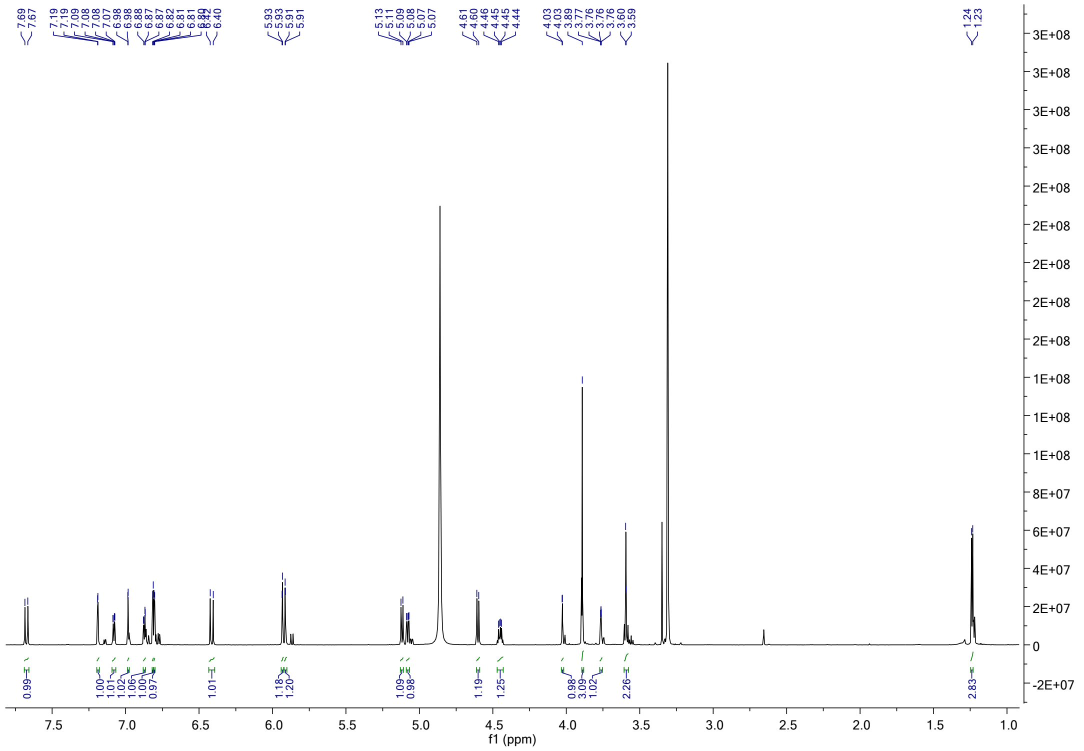

Figure S47. ^{1}H NMR spectrum (CD_3OD, 800 MHz) of compound **7**.

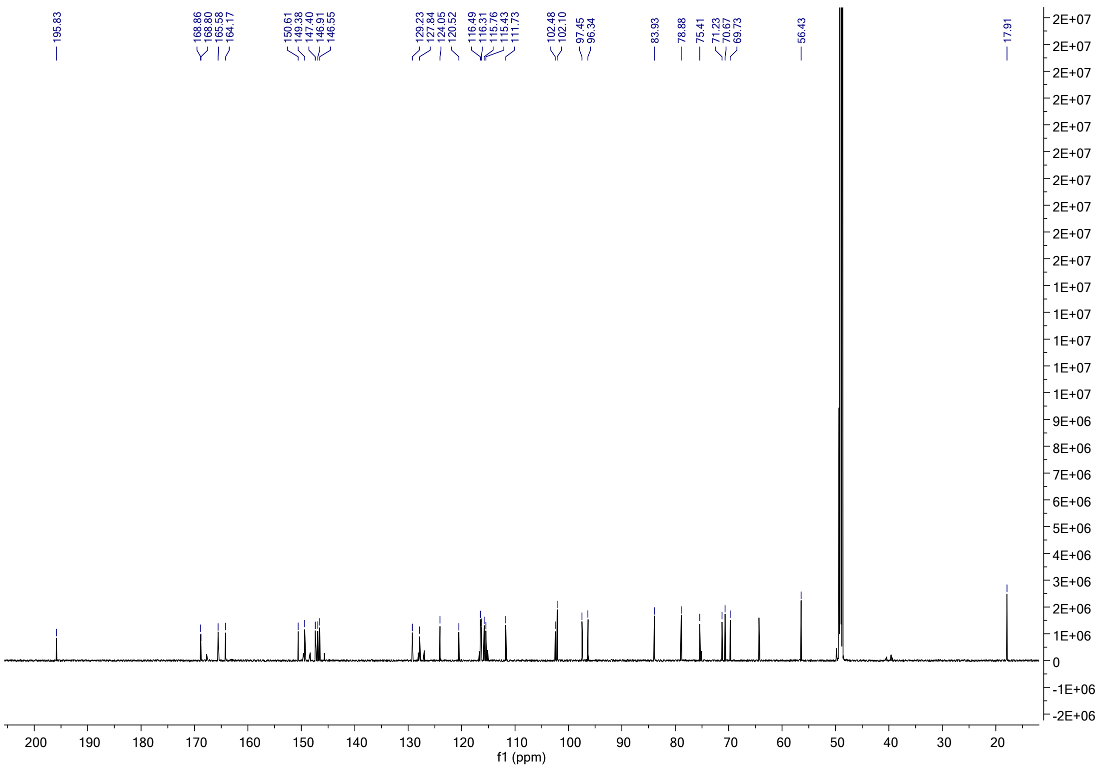

Figure S48. ^{13}C NMR spectrum (CD_3OD, 200 MHz) of compound **7**.

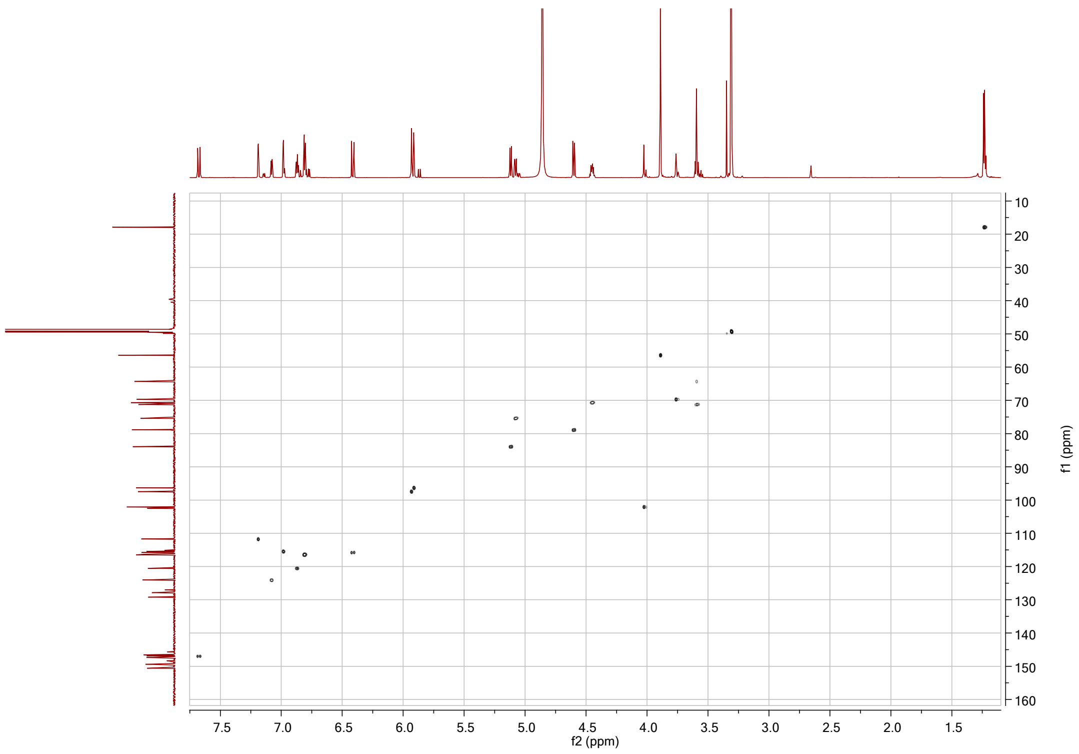

Figure S49. HSQC spectrum (CD_3OD, 800 MHz) of compound **7**.

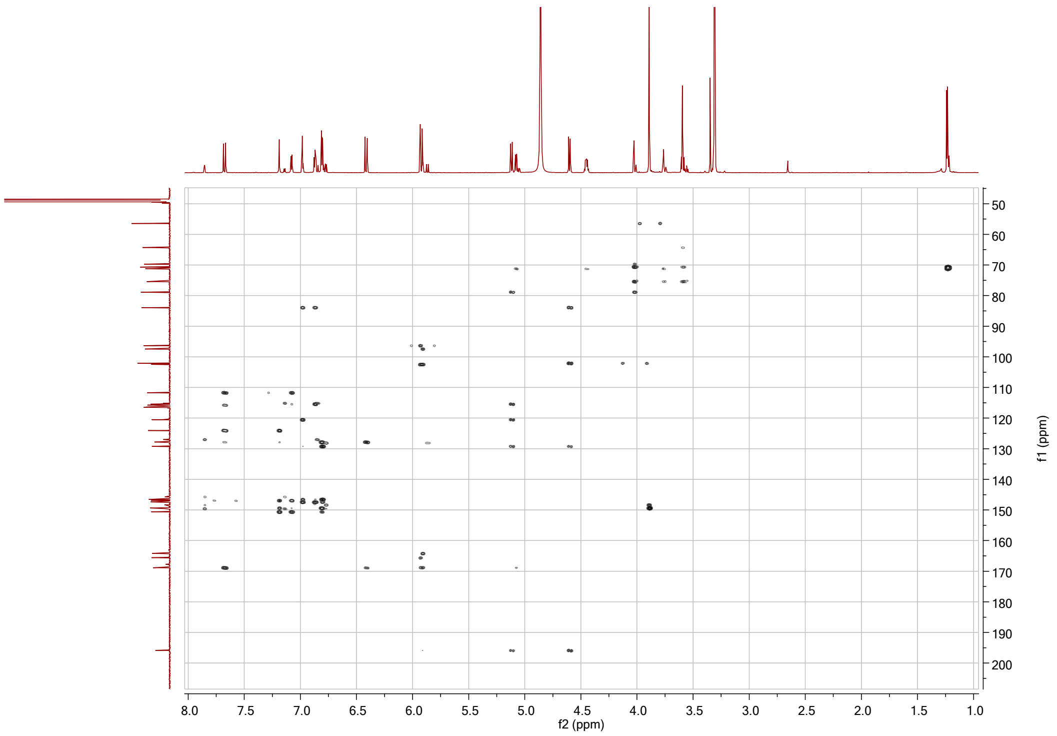

Figure S50. HMBC spectrum (CD_3OD, 800 MHz) of compound **7**.

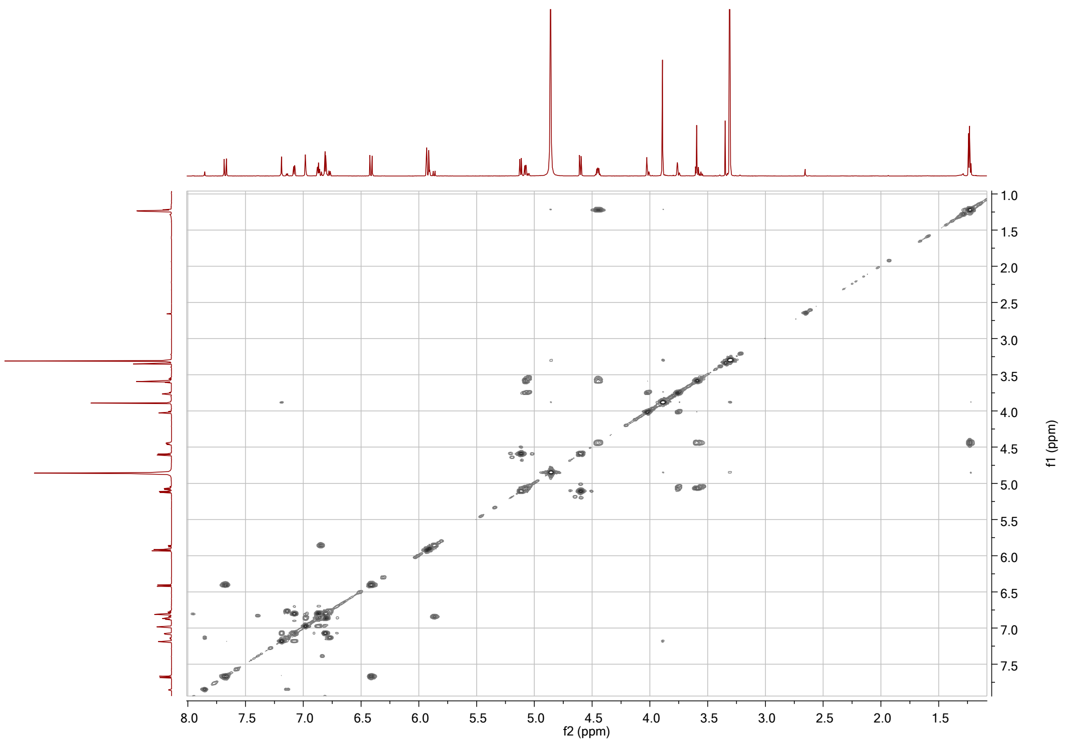

Figure S51. ^1H-^1H COSY spectrum (CD_3OD, 800 MHz) of compound **7**.

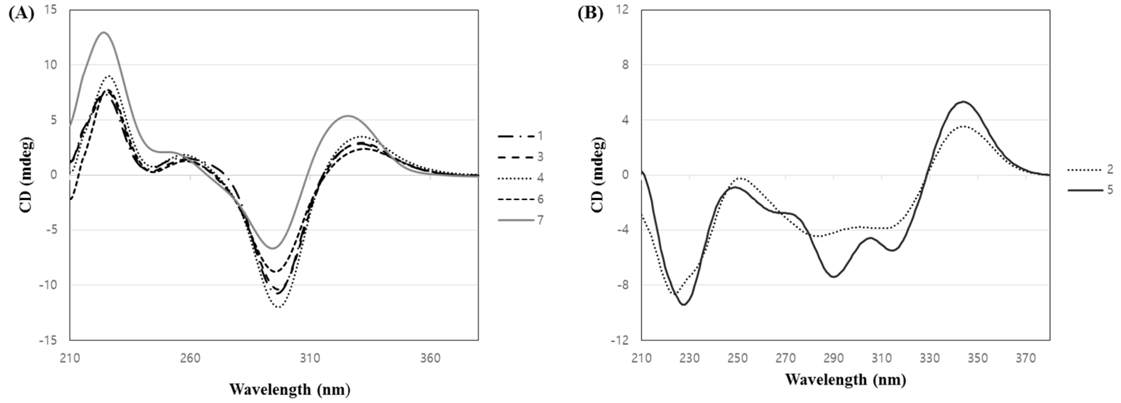

Figure S52. Experimental circular dichroism (ECD) spectra of compounds **1**–**7**. (A) compounds **1**, **3**, **4**, **6** and **7**; (B) compounds **2** and **5**

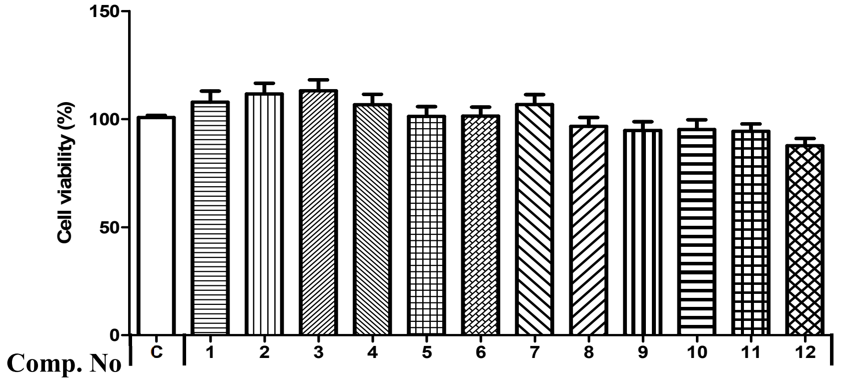

Figure S53. Cytotoxicity of compounds **1**–**12** in HT22 cells. The compounds were treated at 20 μM for 24 h, the cytotoxicity was calculated compared with control group.

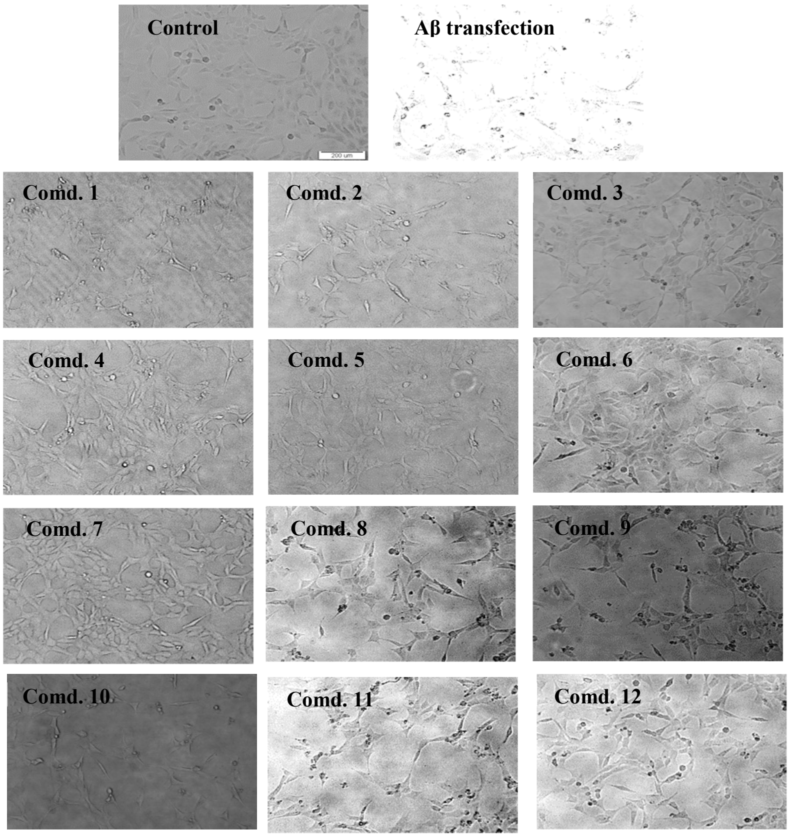

Figure S54. Cell morphology of HT22 cells treated with compounds **1–12** after pEGFP-C1/Aβ_{1-42} plasmid transfection. The cells were transfected with Aβ_{1-42} plasmid using Lipofectamine for 10 h. The transfected cells were exposed to test compound at 20 μM for 24 h.

Table S1. BLAST results of *M. seguinii*.

**GenBank BLAST Report**		
*rbc*L (703 bp)	*matK (1,221 bp)*	*trn*H-*psb*A (495 bp)
Myrsine hermogenesii<br>1299[i] 100%[ii] MG718225.1[iii]	*Ardisia guianensis*<br>2224[i] 99%[ii] JF416280.1[iii]	*Myrsine umbellata*<br>891[i] 99%[ii] KF421096.1[iii]
Myrsine coriacea<br>1293[i] 99%[ii] Z80204.1[iii]	*Myrsine africana*<br>2196[i] 99%[ii] AJ429290.1[iii]	*Myrsine umbellata*<br>891[i] 99%[ii] KF421094.1[iii]
Ardisia guianensis<br>1291[i] 100%[ii] KF981277.1[iii]	*Ardisia polysticta*<br>2174[i] 99%[ii] KC465962.1[iii]	*Myrsine seguinii*<br>880[i] 99%[ii] MF926115.2[iii]
*trn*L-*trn*F (544 bp)	ITS (685 bp)	
Myrsine africana<br>1000[i] 99%[ii] AJ430880.1[iii]	*Myrsine seguinii*<br>1190[i] 98%[ii] KP092669.1[iii]	
Ardisia polysticta<br>985[i] 99%[ii] KC465962.1[iii]	*Myrsine seguinii*<br>1188[i] 98%[ii] MF926239.1[iii]	
Ardisia crenata var. bicolor<br>977[i] 99%[ii] MF926169.2[iii]	*Myrsine chathamica*<br>1181[i] 98%[ii] EF660539.1[iii]	

[i)] Maximum match Scores, [ii)] Maximum similarities. [iii)] Accession Num.
